# HER2 status of CTCs by peptide-functionalized nanoparticles as the diagnostic biomarker of breast cancer and predicting the efficacy of anti-HER2 treatment

**DOI:** 10.3389/fbioe.2022.1015295

**Published:** 2022-09-28

**Authors:** Mengting Wang, Yaxin Liu, Bin Shao, Xiaoran Liu, Zhiyuan Hu, Chen Wang, Huiping Li, Ling Zhu, Ping Li, Yanlian Yang

**Affiliations:** ^1^ CAS Key Laboratory of Standardization and Measurement for Nanotechnology, CAS Key Laboratory of Biological Effects of Nanomaterials and Nanosafety, CAS Center for Excellence in Nanoscience, National Center for Nanoscience and Technology, Beijing, China; ^2^ University of Chinese Academy of Sciences, Beijing, China; ^3^ Key Laboratory of Carcinogenesis and Translational Research (Ministry of Education/Beijing), Department of Breast Oncology, Peking University Cancer Hospital & Institute, Beijing, China

**Keywords:** circulating tumor cells, peptide nanomaterials, HER2 phenotyping, prognosis, breast cancer

## Abstract

Efficacy of anti-human epidermal growth factor receptor 2 (HER2) treatment is impacted by tissue-based evaluation bias due to tumor heterogeneity and dynamic changes of HER2 in breast cancer. Circulating tumor cell (CTC)-based HER2 phenotyping provides integral and real-time assessment, benefiting accurate HER2 diagnosis. This study developed a semi-quantitative fluorescent evaluation system of HER2 immunostaining on CTCs by peptide-functionalized magnetic nanoparticles (Pep@MNPs) and immunocytochemistry (ICC). 52 newly-diagnosed advanced breast cancer patients were enrolled for blood samples before and/or after first-line treatment, including 24 patients who were diagnosed with HER2+ tumors and treated with anti-HER2 drugs. We enumerated CTCs and assessed levels of HER2 expression on CTCs in 2.0 ml whole blood. Enumerating CTCs at baseline could distinguish cancer patients (sensitivity, 69.2%; specificity, 100%). 80.8% (42/52) of patients had at least one CTCs before therapy. Patients with <3 CTCs at baseline had significantly longer progression-free survival (medians, 19.4 vs. 9.2 months; log-rank *p* = 0.046) and overall survival (medians, not yet reached; log-rank *p* = 0.049) than those with ≥3 CTCs. Both HER2+ and HER2-low patients could be detected with HER2 overexpression on CTCs (*CTC*-HER2+) (52.6%, 44.4%, respectively), whereas all the HER2-negative patients had no *CTC*-HER2+ phenotype. Among HER2+ patients with ≥3 CTCs at baseline, objective response only appeared in pretherapeutic *CTC*-HER2+ cohort (60.0%), rather than in *CTC*-HER2– cohort (0.0%) (*p* = 0.034). In conclusion, we demonstrate the significance of CTC enumeration in diagnosis and prognosis of first-line advanced breast cancer, and highlight the value of CTC-HER2 status in predicting efficacy of anti-HER2 treatment.

## Introduction

Breast cancer is responsible for a leading cause of cancer-associated deaths for global females (Siegel et al., 2022). Human epidermal growth factor receptor 2 (HER2) plays a vital role in tumorigenesis, invasion, and metastasis of breast cancer, leading to a poor prognosis and shorter survival (Slamon et al., 1989). Anti-HER2 therapy and combined chemotherapy is recommended as standard first-line treatment for HER2-positive (HER2+) metastatic breast cancer patients according to most guidelines (Gennari et al., 2021; Shead et al., 2022). With more evidence to good efficacy of trastuzumab deruxtecan (DS-8201a) for HER2-low breast cancer patients, HER2-low is becoming important in some guidelines for molecular classification of breast cancer (up to 55%) (Modi et al., 2020; Li and Jiang., 2021; Diéras et al., 2022; Modi et al., 2022). However, a cohort of HER2+ patients or HER2-low patients fails to achieve good outcomes from use of trastuzumab or DS-8201a (30%—67%) (Slamon et al., 2001; Baselga et al., 2012; Diéras et al., 2022). It might be because of HER2 heterogeneity in primary/metastatic tumor sites (Stemmler et al., 2005; Zidan et al., 2005; Niikura et al., 2012). Tissue biopsies by immunohistochemistry (IHC) and/or fluorescence *in situ* hybridization (FISH) have been “gold standard” for diagnosing HER2+ candidates prior to anti-HER2 therapy (Wolff et al., 2007; Wolff et al., 2013). Nevertheless, conventional sampling of tumor tissues through surgery or puncture in metastasis patients is constrained by complicated manipulation, harmful invasion, and difficulty in a comprehensive tumor portrait (e.g., HER2). Therefore, it is necessary to achieve more sensitive and real-time biomarkers for HER2 diagnosis.

A noninvasive blood-based detection using circulating tumor cells (CTCs) is blooming to be a surrogate to histopathologic assessments. CTCs have similar genetic and epigenetic properties to primary/metastatic tumor cells (Plaks et al., 2013). Multiple investigations have been implemented for the clinical significances of CTCs in the early diagnosis, prognostication, and monitoring of solid tumors (Cristofanilli et al., 2004; Cristofanilli et al., 2005; Krebs et al., 2011; Yue et al., 2018; Ebright et al., 2020). CTC enumeration has been a valid staging and prognostic biomarker for breast cancer but lack of significance in predicting the survival of HER2+ patients during anti-HER2 treatment (Giordano et al., 2012; Amin et al., 2017; Heitzer et al., 2019). Exploring the gene/protein portraits of breast cancer CTCs should be complementary to the personalized diagnoses and treatments. HER2 overexpression or amplification on CTCs has been investigated in breast cancer over the last decade. The discordance of HER2 status on tumor tissues and CTCs was present (38%—86%) (Fehm et al., 2007; Fehm et al., 2010; Munzone et al., 2010; Zhang et al., 2016; Chen et al., 2019). Jordan et al. demonstrated a spontaneous interconversion of HER2 phenotypes on CTCs *ex vivo* (Jordan et al., 2016). Hence, HER2 reassessments on CTCs might overcome the spatio-temporal heterogeneity of HER2 expression in breast cancer. However, up to date, there is no widely accepted consensus about the criteria of HER2 phenotyping of enriched CTCs. Thus, it is still significant to develop novel methods for CTC-based HER2 phenotyping.

Efficient enrichment of CTCs is essential for acquiring more CTCs before downstream molecular analysis. Based on the unique biophysical or biochemical properties of CTCs, investigators have established different strategies to capture rare CTCs from circulation blood. *CellSearch*, a system approved by U. S. Food and Drug Administration (FDA), is based on immunomagnetic separation. Large size and low density of antibodies on the surface of magnetic nanoparticles might be primary reasons for its limited detection rate of breast cancer patients with CTCs (22%—70%) and its low number of CTCs from 7.5 ml blood (Allard et al., 2004; Riethdorf et al., 2007; Riethdorf et al., 2010; Liu et al., 2013). Therefore, it remains valuable to explore HER2 phenotyping on CTCs based on a more sensitive detection of CTCs.

Previously, our group has developed an innovative epithelial cell adhesion molecule (EpCAM) recognition peptide-functionalized magnetic nanoparticles (Pep@MNPs) that can be used to efficiently capture rare epithelial CTCs in 2.0 ml whole blood from patients with various solid tumors (Bai et al., 2014; Liu et al., 2017; Yue et al., 2018; Xu et al., 2019; Tan et al., 2021). Pep@MNPs has superior sensitivity (54%—69%) to *CellSearch* in breast cancer patients on account of smaller diameter and higher density of recognition peptides on the surface of magnetic nanoparticles (Bai et al., 2014; Liu et al., 2017; Zhou et al., 2022). Lower producing cost of peptides in Pep@MNPs becomes another more attractive benefit in clinical application as compared to *CellSearch*. In the present study, Pep@MNPs and classical four-color immunocytochemistry (ICC) were applied to detect CTCs and HER2 proteins on CTCs. We detected pretherapeutic blood samples from newly-diagnosed advanced breast cancer patients and monitored second blood samples from HER2+ individuals after anti-HER2 therapy. We consolidated the diagnostic and prognostic values of CTC enumeration with a determined cutoff. Furthermore, evaluation of HER2 status on CTCs was based on a fluorescent semi-quantification evaluation system of HER2 immunostaining. We analyzed the relationship of HER2 expression level on baseline CTCs or its evolution with the efficacy of anti-HER2 therapy.

## Experiments

### Materials

Six cell lines (human breast cancer cell lines SK-BR-3, MDA-MB-453, MCF-7, MDA-MB-468; human mammary epithelial cell line MCF-10A; human histiocytic lymphoma cell U937) were all purchased from American Type Culture Collection (ATCC) (Manassas). EpCAM recognition peptides (VRRDAPRFSMQGLDACGGNNCNN), magnetic nanoparticles and anticoagulation-contained plastic vacuum blood collection tubes were provided by NANOPEP BIOTECH Co. Ltd. (Beijing, China). All the clinical blood samples from human participants in this exploratory study were approved by the institutional review board at the Medical Ethical Committee of Peking Cancer Hospital (No. 2013KT29), and recruited by Department of Breast Oncology, Peking University Cancer Hospital & Institute (Beijing, China).

### Cell culture

Cells were cultured in various basic media supplemented with 10% fetal bovine serum (FBS) and 1% penicillin-streptomycin (Gibco-BRL), including RPMI Medium 1640 (Gibco-BRL) for SK-BR-3 and U937, high-glucose Dulbecco’s Modified Eagle Medium (DMEM) (Gibco-BRL) for MDA-MB-453, MCF-7 and MDA-MB-468, and mammary epithelial cell growth medium (MEGM) kit (Lonza Group Ltd.) for MCF-10A. The cells were maintained at 37°C in an atmosphere with 95% moisture and 5% CO_2_.

### Patients and clinical blood samples

Participants were enrolled from November 2018 to December 2019. The eligible breast cancer patients were newly-diagnosed advanced individuals with normal level of complete blood counts [incl. red blood cells (3.5—5.5*10^12^/L), white blood cells (4.0—10.0*10^9^/L)] (*n* = 52). As a negative control, 10 healthy donors were recruited with no medical history of any serious disease and any surgery within 6 months. Pathologists determined the candidates with positive level of HER2 (HER2+) on tumor tissues (IHC+++; or IHC++ and FISH+), and would recommend anti-HER2 treatments (Wolff et al., 2013). Other patients were diagnosed as non-HER2+, including HER2-negative (HER2–) (IHC0) or HER2-low (IHC+; or IHC++ and FISH–) (Wolff et al., 2013; Tarantino et al., 2020), who were excluded from anti-HER2 treatments. Anti-HER2 therapies in this trial referred to the clinical application of trastuzumab or anti-HER2 tyrosine kinase inhibitor (TKI) (lapatinib, pyrotinib), or a combined use of trastuzumab and anti-HER2 TKI. The Ki67 labeling index (Ki67 LI) on tumor tissues was dichotomized by 14% (Cheang et al., 2009). On the basis of imaging diagnosis and response evaluation criteria in solid tumors (RECIST) (version 1.1) (Eisenhauer et al., 2009), drug response during first-line treatments was monitored for each individual according to their clinical plans, collected by clinicians. Progression-free survival (PFS)/overall survival (OS), was defined from the date of enrollment to the date of clinical progression, death or the last follow-up visit (Eisenhauer et al., 2009). Clinical information from patients was not provided until detection of CTCs and HER2 expression on CTCs were accomplished for all blood samples.

Baseline blood samples were all collected within 30 days before first-line treatment. Second blood draws were conducted in some HER2+ patients after at least two cycles of anti-HER2 therapy. For each blood sample, whole blood was drawn into an anticoagulation-contained plastic vacuum blood collection tube, and delivered to the lab for detection of CTCs and HER2 by Pep@MNPs and ICC within 24 h.

### Construction and characterization of the Pep@MNPs

The Pep@MNPs were constructed through streptavidin-biotin interaction as previously described (Bai et al., 2014). Briefly, with volume ratio (v/v) of 1:2, bare MNPs (5 mg/ml) were incubated with the EpCAM recognition peptide (1 mg/ml) for 60 min at room temperature. Based on magnetic field, washing with phosphate buffer saline (PBS) for three times was needed. Then, the Pep@MNPs were resuspended in PBS (5 mg/ml) and could be stably stored at 4°C. Hydrodynamic diameter and zeta potential of the Pep@MNPs were assessed by dynamic light scattering (DLS) (Zetasizer Nano ZS90, UK) and morphology of the Pep@MNPs was observed on a transmission electron microscope (TEM) (Tecnai G2 20 S-TWIN, United States). Sample preparation strictly complied with the instrument requirements.

### Flow cytometry analysis

5% bull serum albumin (BSA) (Sigma-Aldrich) was used to block the cells for 30 min at 37°C. Cells were respectively incubated with phycoerythrin (PE)-conjugated antibodies for 60 min at 37°C, including anti-EpCAM (BioLegend), anti-HER2 (Sino Biological) and IgG isotype controls (IgG2bk for anti-EpCAM (Biolegend); IgG1 for anti-HER2 (Cell Signaling Technology)). The BD Accuri™ C6 flow cytometer (BD Biosciences) was applied to detect the EpCAM and HER2 immunostaining with PE channel (Ex./Em., 488/574 nm).

### Western blot analysis

Harvested cells were lysed in 1% protease inhibitor-contained Pierce™ IP lysis buffer (Thermo Fisher Scientific, Waltham) on ice for 60 min, followed by high-speed centrifugation (15,000 rpm, 10 min, 4°C). Total proteins were quantified with BCA protein quantification kit (Solarbio) and denatured by 5x SDS-PAGE loading buffer (95°C, 10 min) (CWBIO). Proteins were separated in NUPAGE™ 10% Bis-tris polyacrylamide gels (Invitrogen, Carlsbad) by electrophoresis. Proteins in gels were then transferred onto polyvinylidene fluoride (PVDF) membranes, followed by blocking with 5% nonfat milk solution (w/v) (BD Biosciences) for 2.0 h at room temperature. Primary antibodies [anti-EpCAM (Cell Signaling Technology); anti-HER2 (Cell Signaling Technology); anti-β-actin (EASYBIO)] were then incubated with membranes overnight at 4°C. Horseradish peroxidase (HRP)-conjugated secondary antibodies were used to target primary antibodies for 60 min at room temperature, including horse anti-mouse IgG (Cell Signaling Technology) and goat anti-rabbit IgG (Cell Signaling Technology). Accessible to the ChemiDoc™ MP imaging system (Bio-Rad, Hercules), HRP-labeled proteins (EpCAM/HER2/β-actin) on PVDF membranes were visualized with an enhanced chemiluminescence kit (Thermo Fisher Scientific).

### Detection of HER2 immunostaining on breast cancer cell lines

Pep@MNPs were used to capture breast cancer cells as previously described (Bai et al., 2014). Pep@MNPs were applied to isolate 4’,6-diamidino-2-phenylindole dihydrochloride (DAPI)-pre-stained breast cancer cells (1000 cells/mL) from PBS or to isolate DAPI/DiO-pre-stained breast cancer cells (1000 cells/mL) from spiked system that containing 10^6^ monocyte-like U937 cells. Pep@MNPs were incubated with cells for 60 min at room temperature, followed by magnetic enrichment. After fixation with 2% paraformaldehyde (PFA) for 30 min, captured cells were blocked with 5% BSA solution for 30 min. HER2 on isolated cells was labeled via Alexa Fluor 647-labeled anti-HER2 (BioLegend) (60 min) and detected by an Olympus IX73 fluorescent microscope. All the operations above were performed at room temperature.

### Detection of HER2 immunostaining on CTCs in clinical blood samples

Pep@MNPs and immunocytochemistry (ICC) were used to capture and identify CTCs as previously described (Bai et al., 2014; Liu et al., 2017). Briefly, Pep@MNPs were incubated with 2.0 ml whole blood for 60 min. The magnetically isolated CTCs were then fixed with 2% PFA solution for 30 min and blocked with 5% BSA solution for 30 min. Cytokeratins (CKs)/leukocyte common antigen (CD45)/HER2 were then stained with primary antibodies for 60 min, including Alexa Fluor 488-linked anti-CKs (Abcam), PE-DyLight 594-conjugated anti-CD45 (Abcam), and Alexa Fluor 647-linked anti-HER2 (BioLegend). Cell nuclei were visible via DAPI staining. All the operations above were performed at room temperature. Fluorescence microscope (Olympus IX73) was applied to visualize CTCs and white blood cells (WBC), where CTCs were identified as single cells with DAPI+/CKs+/CD45– and with various levels of HER2 immunostaining.

### Statistical analysis

Based on the software GraphPad Prism (version 8.0), with unpaired Student’s *t*-test, we assessed the differences between two normally-distributed samples. Of note is that if with significantly different variances, unpaired Student’s *t*-test with Welch’s correction was used. With Pearson’s correlation coefficient *r*, we analyzed the correlation between results from two HER2 evaluation methods (e.g., ICC versus (vs.) flow cytometry). We used receiver operating characteristic (ROC) analysis to evaluate the correlation between testing results from two kinds of diagnostic methods (e.g., CTC enumeration vs. clinical HER2 diagnosis). In order to explore the correlations of PFS or OS with CTC number or CTC-based HER2 phenotype at baseline, we employed the Kaplan-Meier plots and utilized the log-rank (Mantel-Cox) test to determine the PFS/OS difference and hazard ratio (HR) between subgroups. In IBM SPSS Statistics software (version 21.0), Fisher’s exact test was used to assess the difference of constituent ratios in contingency tables, such as HER2 status on CTCs vs tumor tissues and objective response rates in patients with various CTC numbers or with various CTC-HER2 subtypes. Fisher’s exact test was chosen, because the minimum of the observed frequencies (*T*_*min*_) was smaller than five (Lucci et al., 2012). Significant level of aforesaid differences and associations can be described by two-tailed *p* values. * *p* < 0.05, ** *p* < 0.01, *** *p* < 0.001 and **** *p* < 0.0001 indicate the statistically significant levels.

## Results

### HER2 detection of breast cancer cells by immunocytochemistry (ICC)

As shown in Supplementary Figure S1A from dynamic light scattering (DLS) analysis, diameter of the Pep@MNPs was around 307.2 nm, slightly larger than that of the unfunctionalized MNPs (approximately 227.8 nm). Zeta potential on Pep@MNPs became more positive than that on unfunctionalized MNPs (** *p* < 0.01) (Supplementary Figure S1A). Images from transmission electron microscope (TEM) showed that structure of MNPs was stably maintained after functionalization (Supplementary Figure S1B). These results demonstrated successful modification of the EpCAM recognition peptides on Pep@MNPs.

**FIGURE 1:**
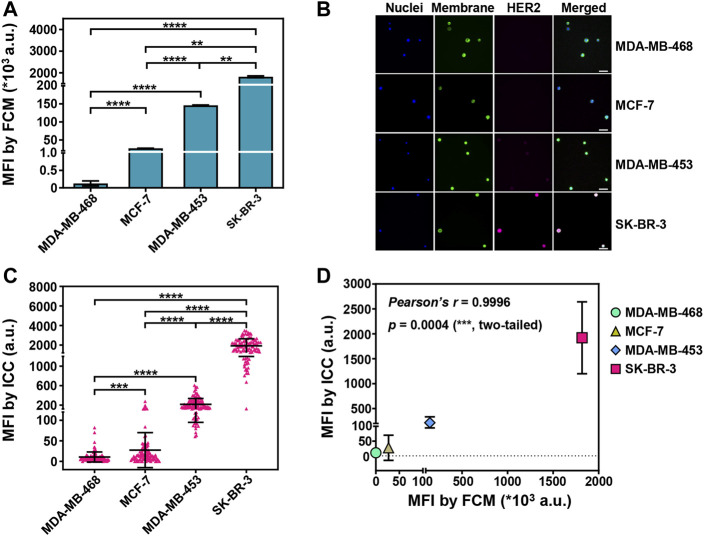
Sensitive detection of HER2 expression on captured breast cancer cells by ICC. **(A)** The mean fluorescent intensities (MFIs) of PE-labeled anti-HER2 on four breast cancer cell lines (SK-BR-3, MDA-MB-453, MCF-7, MDA-MB-468) from flow cytometry (FCM) analysis. Data are presented as means ± SD from 3 parallel samples. **p < 0.01, ****p < 0.0001, unpaired Student’s *t*-test. **(B)** Fluorescent images of the isolated breast cancer cells. The cancer cells were pre-stained with DAPI (blue, nuclei) and DiO (green, membrane), and then the isolated cancer cells were stained with Alexa Fluor 647-conjugated anti-HER2 (pink, HER2). Scale bar: 50 μm. **(C)** The MFIs from HER2 immunostaining on the captured breast cancer cells by Pep@MNPs in System 1 (1,000 breast cancer cells/1 ml PBS). Data are presented as scattered dots from 100 events, together with means ± SD. ****p < 0.0001, ***p < 0.001, unpaired Student’s *t*-test with Welch’s correction. **(D)** Correlation analysis between the MFIs from HER2 immunostaining on four breast cancer cell lines by ICC and by FCM. Pearson’s correlation coefficient *r* = 0.9996, with a significant *p* value (0.0004, ***, two-tailed).

We validated various expression levels of HER2 in four EpCAM-overexpressed breast cancer cell lines by flow cytometry (FCM) and Western blot analysis, the strongest in SK-BR-3, moderate in MDA-MB-453, low in MCF-7, and negative in MDA-MB-468 cells (Figure 1A, Supplementary Figures S2A,B).

With Pep@MNPs and ICC, rare cancer cells were enriched in phosphate buffer saline (PBS) model system (System 1) and HER2 expression was assessed for each breast cancer cell line. The mean fluorescent intensities (MFIs) from HER2 immunostaining were 10-fold higher in SK-BR-3 cells compared to the other cell lines (**** *p* < 0.0001). MDA-MB-453 that had the moderate expression of HER2 exhibited higher MFIs from HER2 immunostaining than MCF-7 and MDA-MB-468 (**** *p* < 0.0001). MCF-7 and MDA-MB-468 that had relatively low expression of HER2 could also be significantly differentiated (*** *p* < 0.001) by HER2 immunostaining (Figures 1B,C). A good correlation between ICC-based and FCM-based HER2 detection was demonstrated (Pearson’s coefficient *r* = 0.9996; *p* = 0.0004, ***) (Figure 1D). In addition, for each breast cancer cell line, rare cancer cells were mixed with 10^6^ EpCAM/HER2-negative U937 cells to better mimic CTCs in complicated blood (System 2). It was nearly the same when comparing the capability of ICC to differentiate HER2 expression between System 1 and 2 (Supplementary Figure S3). These data showed HER2 detection on CTCs by Pep@MNPs and ICC was sensitive.

### Clinical characteristics of breast cancer patients

Fifty-two newly-diagnosed advanced patients were enrolled and documented in Table 1 and Supplementary Table S1. Median age (± standard deviation) was 55 (±11.1) years. Twenty-six individuals (26/52, 50.0%) were diagnosed as poorly-differentiated (G2∼3, G3). All the patients were diagnosed as recurrence disease (44) or primary IV status (8), of whom 35 individuals (67.3%) had visceral metastases. All of the patients were diagnosed by immunohistochemistry (IHC) and/or fluorescence *in situ* hybridization (FISH), including 24 HER2-positive (HER2+) (18 with IHC+++; 6 with IHC++ and FISH+), 13 HER2-low (8 with IHC++ and FISH–; 5 with IHC+), 15 HER2-negative (HER2–) (IHC0). Of the 52 patients, 92.3% (48/52) showed high level of Ki67 LI (over 14%) on tumor tissues. The 24 HER2+ patients accepted first-line anti-HER2 therapies, with trastuzumab and/or combined anti-HER2 tyrosine kinase inhibitor (TKI) (21), or with only anti-HER2 TKI (3). All of non-HER2+ patients (HER2-low or HER2–) had standard first-line therapies without anti-HER2 agents.

**TABLE 1:** Clinical characteristics of recruited breast cancer patients (*n* = 52).

Clinical factors		No. (%)*
Age, years		
Median (± standard deviation)		55 (±11.1)
Range		32–87
Grading		
G1		4 (7.7)
G2		21 (40.4)
G2-3		3 (5.8)
G3		23 (44.2)
Unknown		1 (1.9)
Status of patients		
Recurrence		44 (84.6)
Primary IV		8 (15.4)
Visceral metastasis^a^		
Yes		35 (67.3)
No		17 (32.7)
HER2 status^b^		
HER2+	IHC+++	18 (34.6)
	IHC++, FISH+	6 (11.5)
HER2-low	IHC++, FISH–	8 (15.4)
	IHC+	5 (9.6)
HER2–	IHC0	15 (28.8)
Ki67 LI^c^		
≥14%		48 (92.3)
<14%		4 (7.7)
First-line treatment of 24 HER2+ patients		
With trastuzumab and/or combined anti-HER2 TKI^d^		21 (87.5)
With only anti-HER2 TKI		3 (12.5)

a Metastasis at sites of lung, liver, adrenal gland, brain, kidney, pancreas, peritoneal, pleural effusions, etc.,

b The level of HER2 protein/gene on tumor tissues detected by the immunohistochemistry (IHC)/fluorescence *in situ* hybridization (FISH).

c The percentage of tumor cells that were detected with Ki67 overexpression on tumor tissues, dichotomized by 14%.

d Anti-HER2 tyrosine kinase inhibitor (TKI).

* The number and ratio of individuals with various clinical characteristics in 52 enrolled breast cancer patients.

### Diagnostic significance of baseline CTC enumeration

**FIGURE 2:**
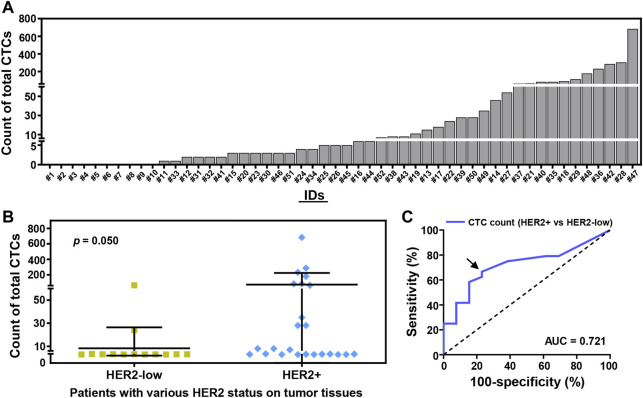
CTC enumeration strongly correlates with the HER2 status of breast cancer patients at baseline. **(A)** Total CTC count in 2.0 ml whole blood from 52 enrolled breast cancer patients (#1–52). 42 (52) of the enrolled patients (80.8%) were detected with ≥1 CTCs/2.0 ml whole blood. **(B)** CTC counting of the patients with various HER2 status on tumor tissues by immunohistochemistry (IHC) and/or fluorescence *in situ* hybridization (FISH), including HER2-low tumor (IHC+; IHC++ and FISH–; *n* = 13), HER2+ tumor (IHC+++; IHC++ and FISH+; *n* = 24). Data are presented as scattered dots, together with means ± SD. *p* = 0.050, unpaired Student’s *t*-test with Welch’s correction. **(C)** Receiver operating characteristic (ROC) curve analysis of CTC enumeration used to detect patients with HER2-low or HER2+ breast tumors. Area under curve (AUC) is presented (0.721 for blue line, *p* = 0.028). Arrow annotates the cutoff of CTC count (>3 CTCs, ≤ 3 CTCs).

Based on Pep@MNPs in 2.0 ml whole blood, CTCs could be detected in 80.8% (42/52) of the enrolled patients before first-line treatments (Figure 2A). There was a broad range of CTC count (0—683 CTCs) at baseline. Baseline CTC enumeration could significantly distinguish breast cancer patients with healthy donors via ROC analysis (area under curve (AUC), 0.875; 95% confidence interval (CI), 0.789–0.961; *p* = 0.0002) (Supplementary Figure S4, Supplementary Table S2). The cutoff of ≥3 CTCs at baseline could detect breast cancer with high sensitivity (69.2%; 95% CI, 55.7%–80.1%) and with remarkable specificity (100%; 95% CI, 72.3%–100%) (Supplementary Table S2). Besides, baseline CTC level in the patients with visceral metastases was significantly higher than those patients without visceral metastases (*p* = 0.022) (Supplementary Figure S5).

After a comparison, there was a certain difference of CTC number between HER2-low patients and HER2+ patients (*p* = 0.050) (Figure 2B). Percentages of patients with baseline CTCs were 69.2% (9/13) in HER2-low cohort, and 79.2% (19/24) in HER2+ cohort (Figure 2B). ROC analysis further demonstrated the cutoff of >3 CTCs at baseline could effectively differentiate HER2-low patients (23.1%, 3/13) from those patients with HER2+ tumors (66.7%, 16/24) (AUC, 0.721; 95% CI, 0.554–0.888; *p* = 0.028) (Figure 2C).

### Semi-quantitative fluorescent evaluation system for HER2 phenotyping on CTCs

**FIGURE 3:**
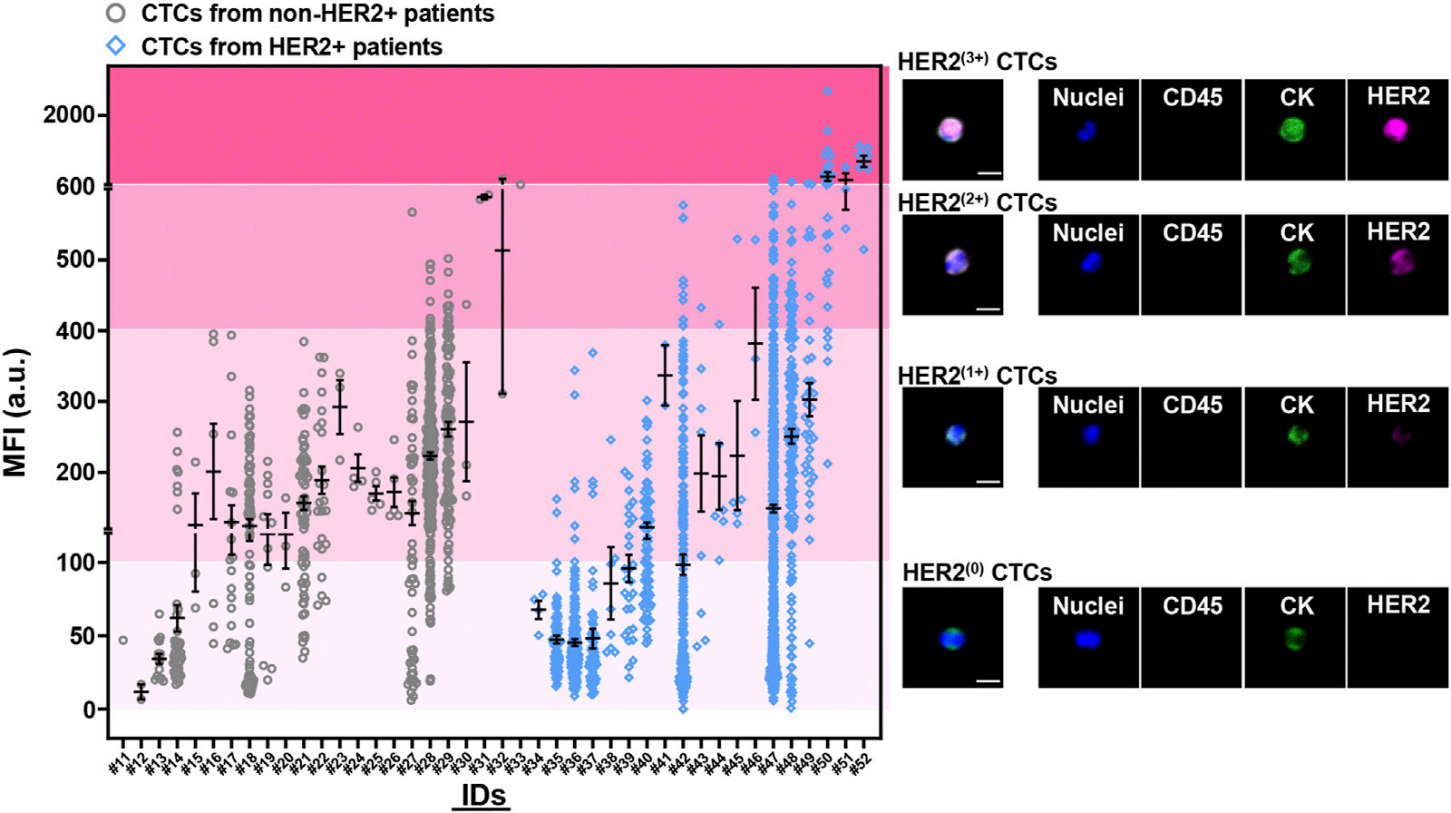
Quantitative analysis of HER2 expression on CTCs from breast cancer patients at baseline. Four levels of HER2 expression on CTCs were developed based on fluorescent quantification (incl. HER2^(0)^ CTCs, HER2^(1+)^ CTCs, HER2^(2+)^ CTCs, HER2^(3+)^ CTCs). Left, mean fluorescent intensities (MFIs) from HER2 immunostaining on enriched CTCs for each patient (#11–52) at baseline. Patients who had baseline CTCs were presented as two cohorts, with non-HER2+ tumor (IHC0; IHC+; IHC++ and FISH–) (gray, #11–33; *n* = 23), or with HER2+ tumor (IHC+++; IHC++ and FISH+) (blue, #34–52; *n* = 19). Data are presented as scattered dots, together with means ± standard error of mean (SEM). *x* axis, the IDs of patients. HER2 expression on CTCs were quantitatively determined as four MFI ranges from HER2 immunostaining, including no staining/barely perceptible staining (HER2^(0)^; MFI 0—100 a.u.), faint staining (HER2^(1+)^; MFI 100—400 a.u.), moderate staining (HER2^(2+)^; MFI 400—600 a.u.), strong staining (HER2^(3+)^; MFI >600 a.u.). Right, the representative fluorescent graphs of the captured CTCs with different levels of HER2 expression. Scale bar: 10 μm.

Quantitative analysis of MFIs from HER2 immunostaining on the enriched CTCs was performed to establish a rational system for HER2 phenotyping on CTCs. On the basis of the difference of HER2 expression on single CTCs between patients with or without HER2+ breast tumors (HER2+ vs. non-HER2+), we defined four subpopulations of CTCs with various MFI ranges of HER2 immunostaining, including HER2^(0)^ CTCs (MFI 0—100 a.u.), HER2^(1+)^ CTCs (MFI 100—400 a.u.), HER2^(2+)^ CTCs (MFI 400—600 a.u.) and HER2^(3+)^ CTCs (MFI >600 a.u.) (Figure 3). There was an overlap of the MFIs of HER2 immunostaining on CTCs between HER2+ patients and non-HER2+ patients, suggesting the importance of an additional statistical cutoff. Herein, the cutoff of HER2^(2+)^ CTC ratio (12%) was proposed by ROC analysis of HER2 immunostaining on CTCs between IHC0/+ patients and IHC++ patients (Supplementary Figure S6). Therefore, HER2 overexpression on CTCs (*CTC*-HER2+) was determined as the case with >12% of HER2^(2+)^ CTCs or at least one HER2^(3+)^ CTCs in total CTCs.

Among the patients with CTCs at enrollment, 10 (19) of HER2+ patients (52.6%) were identified as *CTC*-HER2+ and 19 (23) of non-HER2+ patients (HER2-low/HER2–) (82.6%) were *CTC*-HER2– (Table 2, Supplementary Figure S7), showing an overall coincidence rate of 69.0% (29/42). In non-HER2+ patients, 4 (9) (44.4%) of HER2-low individuals were detected with *CTC*-HER2+ at baseline, which was not observed in HER2– patients (0/14, 0.0%). We demonstrated a statistical difference between the HER2 status on CTCs and on tumor tissues [Kappa’s test, *κ* = 0.191 (*p* = 0.012); Fisher’s exact test, *p* = 0.002].

**TABLE 2:** Comparison of HER2 assessments on CTCs and tumor tissues from breast cancer patients at baseline.

	Tumor tissue-based		
CTC-based	HER2–, no. (%)*	HER2-low, no. (%)*	HER2+, no. (%)*
≥1 CTCs	14	9	19
*CTC-*HER2–^a^	14 (100)	5 (55.6)	9 (47.4)
*CTC-*HER2+^b^	0 (0.0)	4 (44.4)	10 (52.6)

*κ* = 0.191 (Kappa’s test) (*p* = 0.012); Fisher’s exact test, *p* = 0.002.

a The HER2-negative phenotype on CTCs, among the patients with ≥1 CTCs at baseline, determined as the case without >12% HER2^(2+)^ CTCs and without HER2^(3+)^ CTCs.

b The HER2-positive phenotype on CTCs, among the patients with ≥1 CTCs at baseline, determined as the case with >12% HER2^(2+)^ CTCs or with HER2^(3+)^ CTCs.

* The number and percentage of the cases with various *CTC*-HER2 phenotypes (*CTC*-HER2– or *CTC*-HER2+) in the patients with different molecular subtypes from tumor tissues.

### Correlation of CTC number and CTC-HER2 status with patient survival

**FIGURE 4:**
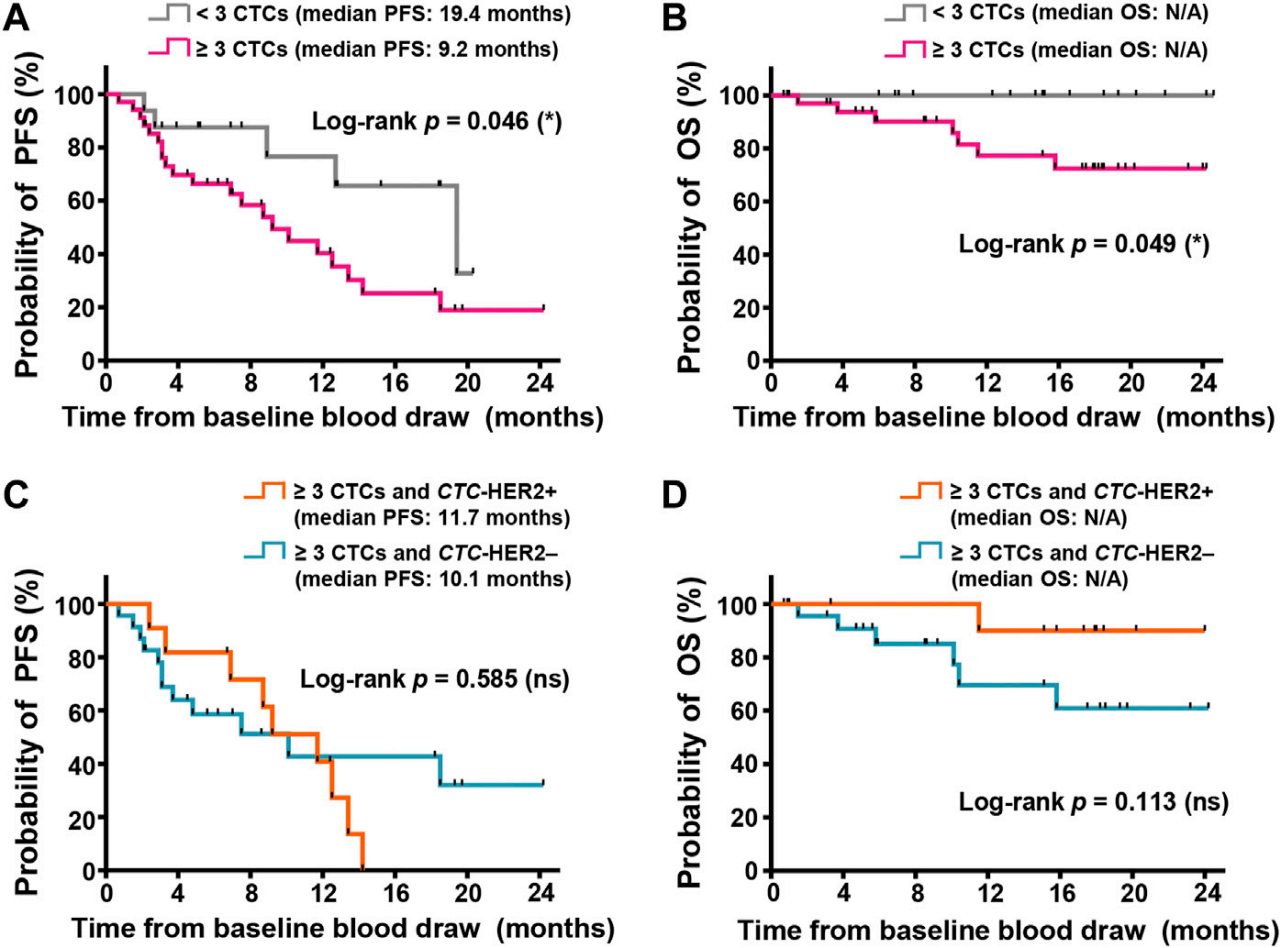
CTC enumeration and HER2 phenotyping on CTCs might correlate with survivals of advanced breast cancer, independent of molecular subtypes. **(A,B)** Kaplan-Meier plots of progression-free survival (PFS) **(A)** and overall survival (OS) **(B)** in the patients with various CTC enumeration (≥3 CTCs in pink, < 3 CTCs in gray) from 2.0 ml whole blood at baseline. In PFS **(A)**, log-rank *p* = 0.046, hazard ratio (HR) of progression (<3 CTCs vs. ≥ 3 CTCs) = 0.387 (95% CI, 0.175–0.854); in OS **(B)**, log-rank *p* = 0.049; both are <0.05 (significant, *). **(C,D)** Kaplan-Meier plots of PFS **(C)** and OS **(D)** in the cohort with ≥3 CTCs and various CTC-based HER2 status (*CTC*-HER2+ in orange, *CTC*-HER2– in dark green) from 2.0 ml whole blood at baseline. In PFS **(C)**, log-rank *p* = 0.585 (not significant, ns). In OS **(D)**, *p* = 0.113 (ns), HR of death (*CTC*-HER2+ vs. *CTC*-HER2–) = 0.215 (95% CI, 0.048–0.959). N/A, median OS not yet reached.

Among totally enrolled 52 patients, we observed less CTCs (<3 CTCs) at baseline (*n* = 16) had significantly longer progression-free survival (PFS) (medians, 19.4 vs. 9.2 months) (*p* = 0.046; hazard ratio (HR) of progression = 0.387; 95% CI of HR, 0.175–0.854) and overall survival (OS) (medians, not yet reached) (*p* = 0.049), as compared to high CTC number (≥3 CTCs) at baseline (*n* = 36) (Figures 4A,B). In the patients with ≥3 CTCs, individuals were divided into subgroups with various CTC-HER2 phenotypes. There’s no significant difference of PFS between *CTC*-HER2+ patients and *CTC*-HER2– patients (Figure 4C). An impressive result was achieved in OS analysis, where patients with *CTC*-HER2+ phenotype at baseline exhibited a slightly better OS compared with *CTC*-HER2– patients at baseline (medians, not yet reached) (*p* = 0.113; HR of death = 0.215; 95% CI of HR, 0.048–0.959) (Figure 4D).

For patients with various molecular subtypes, there was no strong correlation of CTC status at enrollment with survivals in non-HER2+ individuals, and also in HER2+ individuals (Supplementary Figures S8, S9). According to pretherapeutic CTC-HER2 status in HER2+ patients who were detected with ≥3 CTCs, we observed that, median PFS of *CTC*-HER2+ patients was 9.2 months, longer than 4.8 months in *CTC*-HER2– cohort, but not reaching an overall significant difference (*p* = 0.674) (Supplementary Figure S9C). Meanwhile, we demonstrated that pretherapeutic *CTC*-HER2+ patients were more possible to achieve longer OS than patients with *CTC*-HER2– phenotype (medians, not yet reached) (*p* = 0.101; HR of death = 0.187; 95% CI of HR, 0.024–1.44) (Supplementary Figure S9D).

### Predictive significance of CTC number and CTC-HER2 status for drug response to anti-HER2 therapy

HER2+ patients (*n* = 24) were all treated with anti-HER2 regimes after enrollment. Best overall responses of 22 individuals were available (22/24), retrospectively documented in Supplementary Table S1. 11 (22) of the individuals (50.0%) achieved objective response [complete response (CR) or partial response (PR)]. 40.9% of patients (9/22) were stable disease and 9.1% of patients (2/22) were progressive disease. Hence, this trial indicated an objective response rate (ORR) of 50.0% and a disease control rate of 90.9% after anti-HER2 therapy.

As shown in Table 3, 83.3% (5/6) of the patients with <3 CTCs at enrollment benefited from anti-HER2 therapy, and 37.5% (6/16) of the patients with ≥3 CTCs had objective response (CR or PR) as well (*p* = 0.149). We further investigated the predictive value of CTC-based HER2 phenotyping at baseline among the patients with ≥3 CTCs. The objective response only appeared in *CTC*-HER2+ cohort (6/10, 60.0%), rather than in *CTC*-HER2– cohort (0/6, 0.0%), with a statistically significant difference (*p* = 0.034) (Table 3).

**TABLE 3:** Correlation analysis of baseline CTC enumeration or *CTC*-HER2 phenotyping with objective response after anti-HER2 therapy.^a^

CTC-based variable	CR + PR, no. (%)	*p* value
Total CTCs in 22 HER2+ patients		
<3 CTCs (*n* = 6)	5 (83.3)	0.149
≥3 CTCs (*n* = 16)	6 (37.5)	
*CTC*-HER2 in 16 HER2+ patients with ≥3 CTCs		
*CTC*-HER2– (*n* = 6)	0 (0.0)	0.034^b^
*CTC*-HER2+ (*n* = 10)	6 (60.0)	

a Totally 22 (24) of HER2+ patients were included in this analysis, because they accepted first-line anti-HER2 treatment and were provided with best overall responses. On basis of RECIST 1.1, objective response rate (ORR) was calculated as the percentage of the cases with complete response (CR) or partial response (PR) in the patients with various CTC number or CTC-HER2 phenotype at baseline. Fisher’s exact *p* values were applied to assess the correlations.

b Indicates a significant level of correlation (*p* < 0.05).

**FIGURE 5:**
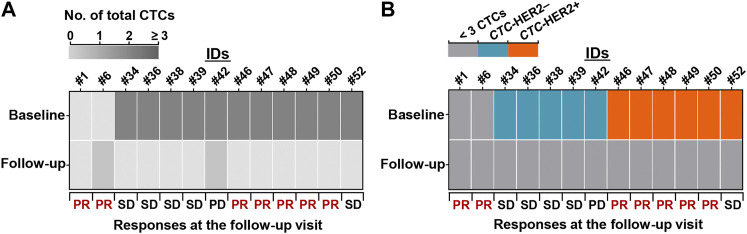
Reduction of CTC number and pretherapeutic *CTC*-HER2+ might predict patient’s benefit from anti-HER2 treatment. **(A,B)** Heatmaps of dynamic changes of total CTC count **(A)** and *CTC*-HER2 phenotype **(B)** from baseline to follow-up visit. 13 HER2+ patients with second blood draws and clinical responses at the follow-up visit were presented.

Of 24 HER2+ patients, 13 patients had second blood samples after several cycles of therapy (2–5 cycles). The CTC detection and clinical response at follow-up visit were collected in Supplementary Table S1. Eleven of 13 patients (84.6%) achieved the CTC decrease (from ≥3 CTCs to <3 CTCs), and the other two patients were maintained with a low level of CTC number (<3 CTCs) (Figure 5A). 5 (11) (45.5%) of patients with CTC decrease and 2 (2) (100%) of patients with sustainable <3 CTCs achieved a response of PR at follow-up visit.

Among the 11 patients with CTC decrease, 6 individuals were *CTC*-HER2+ at baseline, 5 individuals were *CTC*-HER2– at baseline (Figure 5B). Intriguingly, we observed, 83.3% (5/6) of pretherapeutic *CTC*-HER2+ patients had a follow-up response of PR, whereas none of 5 pretherapeutic *CTC*-HER2– patients responded with PR at follow-up visit (*p* = 0.015) (Figure 5B).

## Discussion

This work aimed to explore the values of CTC enumeration and HER2 phenotyping on CTCs by Pep@MNPs and ICC in the diagnosis, prognosis and prediction of newly-diagnosed advanced breast cancer.

With the advantages of peptides and larger magnetic nanoparticles of Pep@MNPs, 80.8% of breast cancer patients were detected with baseline CTCs (0–683 CTCs per 2.0 ml whole blood) in this trial, superior to other reports by *CellSearch* (45%—70%; commonly several to tens of CTCs per 7.5 ml whole blood) (Cristofanilli et al., 2005; Riethdorf et al., 2007; Baccelli et al., 2013; Liu et al., 2013; Plaks et al., 2013). High sensitivity (69.2%) and specificity (100%) of CTC enumeration by Pep@MNPs between patients and healthy donors were validated in our trial. It could be explained by excellent targeting sensitivity of our Pep@MNPs to the EpCAM on CTCs without interference from numbers of background blood cells (Bai et al., 2014). High CTC level by our Pep@MNPs at baseline was demonstrated to significantly correlate with visceral metastasis (*p* = 0.022). It may be because blood-based metastatic spread to distant sites requires enough CTCs (Giuliano et al., 2014).

As one of the classical immunohistochemical biomarkers used to determine the molecular subtypes of breast cancer, HER2 overexpression is an indicator of strong proliferation and invasion of tumor cells (Jordan et al., 2016). Nonetheless, survivals of HER2+ patients can be significantly improved by anti-HER2 treatment, as compared with triple-negative breast cancer patients (Giordano et al., 2012). With more evidence showing the possibility of anti-HER2 therapy in HER2-low breast cancer, investigators are paying more attention to distinct features of HER2-low breast tumors. Zhang et al. (2022) demonstrated that HER2-low patients had a significantly lower level of Ki67 expression than both HER2-negative and HER2+ patients. In our trial, we particularly demonstrated that HER2-low patients were detected with less baseline CTCs than that of HER2+ patients (*p* = 0.050, Figure 2B). Hence, CTC enumeration by Pep@MNPs could effectively indicate the unique property of HER2-low patients, with good concordance to low proliferation of their breast tumors.

Our group previously reported the pretherapeutic CTC enumeration by Pep@MNPs with the cutoff of 3 CTCs per 2.0 ml whole blood could predict the PFS and OS of metastatic breast cancer patients after first-line chemotherapy (Liu et al., 2017). Other published works by *CellSearch* have validated the best overall prognosis of metastatic breast cancer with new-line therapy using the cutoff of 5 CTCs per 7.5 ml blood (Cristofanilli et al., 2004; Cristofanilli et al., 2005; Giordano et al., 2012; Cristofanilli et al., 2019). In this trial, our data suggested that regardless of molecular subtypes of newly-diagnosed advanced breast cancer, patients with less than 3 baseline CTCs had a significantly longer PFS (*p* = 0.046) and OS (*p* = 0.049) than patients with ≥3 CTCs at enrollment (medians of PFS, 19.4 vs. 9.2 months; medians of OS, not yet reached). Therefore, we strengthen the prognostic value of Pep@MNPs-based CTC enumeration in newly-diagnosed advanced breast cancer independent of histopathological subtypes. It is also worth noting that, no significant difference of PFS and OS was observed in HER2+ patients with various levels of baseline CTC number in the present study. As described by other retrospective trials, indeed, pretherapeutic CTC number was not a necessary biomarker to predict the survivals of patients with anti-HER2 treatment (Giuliano et al., 2011; Giordano et al., 2012; Zhang et al., 2016; Deutsch et al., 2020). We speculated that special interaction between CTCs and anti-HER2 drugs might be a reason for the unsatisfactory prognostic value of CTC enumeration in HER2+ patients.

To investigate the clinical significance of HER2 expression on CTCs after Pep@MNPs-based isolation, we first established a reasonable multilevel system for CTC-based HER2-positive (*CTC*-HER2+). We determined those patients with a high ratio of HER2-moderately-stained CTCs (>12%) or at least one HER2-strongly-stained CTCs as *CTC*-HER2+. Previous studies were extensively based on *CellSearch* and ICC. Some investigators defined the positive level of HER2 on CTCs with at least one HER2-strongly/moderately stained CTCs (Fehm et al., 2010; Riethdorf et al., 2010; Beije et al., 2016; Wang et al., 2020). Similar to our study, other trials also explored the contribution of HER2-positive CTC ratio, such as 30% or 50% as the cutoffs (Pestrin et al., 2009; Liu et al., 2013; Zhang et al., 2016). The difference of these criteria for detecting CTC-based HER2 positive might be associated with the difference in sensitivity of detection, various inclusion characteristics of patients, thereby closely associated with clinical values of HER2 status on CTCs.

Prognostication of survivals by HER2 phenotyping on CTCs was investigated in our trial. In patients with worse prognosis by pretherapeutic CTCs (i.e., ≥ 3 CTCs, with a significantly shorter PFS/OS than <3 CTCs), we didn’t identify individuals with a significantly shorter PFS based on CTC-based HER2 phenotyping at enrollment. According to the trial from Munzone et al. (2010), between the patients with or without CTC-HER2 overexpression, it also showed no visible difference of median PFS (15 vs. 20 weeks). However, other investigators commonly proposed significant correlations of CTC-HER2 status with PFS of HER2+ patients after new-line anti-HER2 therapies (Liu et al., 2013; Zhang et al., 2016). Different therapy lines and regimes of enrolled HER2+ patients might be the primary reasons. Nonetheless, we observed some difference of median PFS between subgroups of CTC-HER2 status (positive vs. negative, 9.2 vs. 4.8 months) in HER2+ patients (Supplementary Figure S9). Thus, in this small sample size, HER2 overexpression on baseline CTCs is not a reliable predictive biomarker for PFS in overall patients or in HER2+ patients after first-line treatment, but might be useful for early detection of tumor progression after anti-HER2 treatment.

Few reports suggest significant correlations of HER2 status on baseline CTCs with patient’s OS after anti-HER2 therapy. Interestingly, among the patients with ≥3 CTCs, we demonstrated that CTC-HER2 phenotyping was relatively correlated with patient’s OS regardless of molecular subtypes. Patients with *CTC*-HER2+ at baseline showed a certain better OS as compared to baseline *CTC*-HER2– cohort (hazard ratio of death, 0.215; 95% CI, 0.048–0.959) (Figure 4D). We validated the predictive value of OS was mainly from patients with HER2+ tumors. Hazard ratio of death risk between pretherapeutic *CTC*-HER2+ and *CTC*-HER2– patients could reach 0.187 (95% CI, 0.024–1.44) (Supplementary Figure S9D). HER2+ patients were treated with anti-HER2 therapies after enrollment. As well known, HER2-targeted therapy can improve the prognosis of HER2+ patients (Giordano et al., 2012). According to our data, negative expression of HER2 on baseline CTCs might represent a more aggressive subtype from HER2+ patients and was easily resistant to the interference with anti-HER2 drugs, thereby possibly guiding a more effective treatment on these patients with combination regimes (e.g., anti-HER2 plus other systemic therapies).

For relapsed metastatic patients in our trial, the time from surgery of primary tumors to baseline blood draws was in range from 1 year to 15 years. Due to hard sampling from metastatic sites, a large number of enrolled patients (63%) were still guided by HER2 status of primary sites when clinicians determined first-line anti-HER2 treatments. Niikura et al. (2012) illustrated that patients with recurrence had more possibility to show HER2 discordance between primary and metastatic sites than those newly-diagnosed metastatic patients (26% vs. 12%). Hence, the biased and invasive tumor biopsies may cause the misclassification of HER2+ patients and overtreat the patients from anti-HER2 treatment. Evolutionary HER2 status on breast tumors underlines novel noninvasive biomarkers that reflect real state of tumor and its HER2 expression, represented by CTCs and HER2 proteins on CTCs.

It is of importance to explore whether CTC enumeration and CTC-HER2 phenotyping by Pep@MNPs system at enrollment are able to predict the objective response of HER2+ tumors to anti-HER2 therapy. On one hand, we demonstrated that HER2+ patients with less CTCs at baseline (<3 CTCs) had a slightly higher objective response rate (ORR) (83.3%) than those HER2+ patients with ≥3 baseline CTCs (37.5%). Indeed, to the best of our knowledge, few studies show the prediction of objective response with baseline CTC number by *CellSearch*. Wallwiener’s group detected baseline CTCs from 15 patients before first-line anti-HER2 therapy, whereas no significant difference was shown between the rates of PD in two subgroups (≥5 CTCs vs. < 5 CTCs, 14.3% vs. 25.0%) (Deutsch et al., 2020). On the other hand, in patients with ≥3 baseline CTCs, we remarkably confirmed that patients with *CTC*-HER2+ phenotype at baseline were more likely to benefit from anti-HER2 antibodies than *CTC*-HER2– individuals (ORR, 60.0% vs. 0.0%; *p* = 0.034). According to the theory of Jordan et al. (2016), HER2-negative CTCs were insensitive to anti-HER2 antibodies and easily resistant to cytotoxic chemotherapy. *CTC*-HER2+ patients might have more HER2-overexpressed cancer cells in tumor lesions, thereby more sensitive to anti-HER2 agents. Therefore, to some extent, our data indicated that enumerating CTCs by Pep@MNPs at baseline tends to early discriminate aggressive or indolent HER2+ breast tumors before treatment with anti-HER2 antibodies. Additional HER2 assessment on baseline CTCs could significantly improve the power of CTCs to forecast efficacy of anti-HER2 antibodies.

Recently, the present HER2 receptors on HER2-low tumor cells initiate the global attentions to the feasibility of anti-HER2 strategies in HER2-low breast cancer. Several great clinical trials that using trastuzumab-deruxtecan (DS-8201a) have initially shared good news with the unresectable/metastatic HER2-low patients, especially in the latest DESTINY-Breast04 phase 3 trial, HER2-low patients with DS-8201a had a significantly better PFS and OS than those with physician’s choice of chemotherapy (medians of PFS, 9.9 vs. 5.1 months; medians of OS, 23.4 vs. 16.8 months) (Modi et al., 2020; Diéras et al., 2022; Modi et al., 2022).

HER2-low phenotyping by IHC maybe only represent a portion of tumor HER2 portrait. CTCs that are considered as potential metastatic “seeds” are blooming to provide complementary HER2 status. Our study demonstrated that tissue-based HER2-low patients had a certain possibility (4/9, 44.4%) to be detected with HER2 overexpression on CTCs before therapy, similar to tissue-based HER2+ patients (10/19, 52.6%). Detection of *CTC*-HER2+ may be a possible indicator for the good outcome of DS-8201a superior to classical chemotherapy in HER2-low patients. CTC-based HER2 phenotyping in HER2-low patients with DS-8201a therapy is worthy of being retrospectively or even prospectively investigated in the future.

Clinically, imaging diagnosis is commonly used to monitor the progression of solid tumors regularly during a new-line therapy. Nevertheless, radiographic assessment is inevitably limited by imaging resolution, especially for undetectable tumor lesions. Tumor size-based evaluation combined with additional biologic analysis could better imply tumor progression. Hayes’s group confirmed that, in patients with or without progression disease by imaging detection, CTC enumeration after 4 weeks of treatment were significantly associated with patient’s OS (Budd et al., 2006). Moreover, previous studies have shown that dynamic change of HER2 expression on CTCs after HER2-targeted therapy obviously correlated with resistance and prognosis (Li et al., 2018; Wang et al., 2020). Thus, longitudinal monitoring of CTC number and CTC-HER2 subtype might early forecast the drug response to anti-HER2 treatment (Budd et al., 2006; Li et al., 2018; Wang et al., 2020).

In our study, in HER2+ patients, we monitored the change of CTC number and CTC-HER2 subtype from baseline to follow-up visit. All of the patients showed a CTC decrease (from ≥3 CTCs to <3 CTCs) or a sustainable low level of CTC number (<3 CTCs). The considerable effect of HER2-targeted therapy on high level of epithelial CTC number was concordant with previous findings from other groups, suggesting that elimination of CTCs by trastuzumab is undisputed in HER2+ patients (Bozionellou et al., 2004; Barok et al., 2008; Giuliano et al., 2011; Deutsch et al., 2020). However, most of preclinical or clinical trials provide encouraging proofs to clinical values of early CTC clearance during first-line chemotherapy or other systematic therapies among HER2-negative patients, including the ongoing DETECT IVb trial (Bidard et al., 2014; Wallwiener et al., 2014; Schramm et al., 2016; Deutsch et al., 2020). In the current study, of the patients with CTC number reduced or maintained to less than 3 CTCs, 53.8% individuals could objectively respond to anti-HER2 treatment, and 92.3% individuals reached disease control at follow-up visit. Accordingly, our trial showed that early CTC decrease, monitored by Pep@MNPs, might be promising for early prediction of the impact of anti-HER2 antibodies.

More specially, our data showed, in the patients who were detected with loss of high level of CTCs and *CTC*-HER2+, 83.3% individuals achieved PR at follow-up visit, superior to simply monitoring CTC decrease (45.5%). The intriguing observation underlines that, with HER2 re-assessment on CTCs, we could more deeply understand different biological behaviors of HER2+ tumors during anti-HER2 therapy. Wallwiener et al. (2014) illustrated the reduction of rate of *CTC*-HER2+ patients after 4 weeks of first-line anti-HER2 treatment, but had no comments on its clinical value of predicting therapeutic outcome. Some suggestions from Wang et al. (2020) are in line with this trial, even though they focused on a cohort of HER2-negative patients who was determined as high level of HER2 expression on baseline CTCs (“cHER2+”). Those pretherapeutic cHER2+ patients could achieve partial response with a complete elimination of HER2+ CTCs after the addition of anti-HER2 agents into their therapy regimes (Wang et al., 2020). Therefore, CTC-HER2 follow-up might be applied to provide real-time suggestions for decision-making of anti-HER2 therapies, thereby more effectively administrating the treatment of breast cancer patients.

A few limitations should also be concerned in the present study. From CTC detection and CTC-HER2 phenotyping in newly-diagnosed advanced breast cancer, we illustrated aforesaid remarkable clinical implications. However, this trial has some limitation in a small sample size, especially a small cohort of patients treated with first-line anti-HER2 therapies. A long and more regular follow-up of CTC enumeration and HER2 assessment on CTCs during anti-HER2 treatment should also be an interesting subject in the future. If so, more rigorous thoughts about the change of CTC number and *CTC*-HER2+ subtype could be uncovered for breast cancer, important for the decision making through CTC-based personalized longitudinal evaluations.

## Conclusion

Pep@MNPs can achieve a sensitive detection of CTCs from 2.0 ml whole blood. Less CTCs at baseline indicates a significantly better prognosis than high CTC status. A cohort of HER2-low patients can be detected with *CTC*-HER2+. For HER2+ patients with ≥3 baseline CTCs, *CTC*-HER2+ patients at baseline are significantly easier to benefit from anti-HER2 treatment than *CTC*-HER2– patients. Based on sequential detection, reduction of CTC number and pretherapeutic *CTC*-HER2+ subtype synergistically suggests a real-time benefit from anti-HER2 treatment. Our observations preliminarily demonstrate that HER2 assessment on CTCs perhaps overcomes spatio-temporal heterogeneity of HER2 status by tumor biopsies and more precisely tailors anti-HER2 treatment.

## Data Availability

The original contributions presented in the study are included in the article/Supplementary Material, further inquiries can be directed to the corresponding authors.

## Supplementary material

SUPPLEMENTARY FIGURE S1 Characterization of the Pep@MNPs. **(A)** Hydrodynamic diameters and zeta potentials of magnetic nanoparticles (MNPs) modified with or without a EpCAM recognition peptide (Pep@MNPs; unfunctionalized MNPs) from dynamic light scattering (DLS) analysis. Data were presented as means ± SD from 3 parallel samples. ***p* < 0.01, unpaired Student’s *t*-test with Welch’s correction. **(B)** Transmission electron microscope (TEM) characterization of Pep@MNPs and unfunctionalized MNPs. Scale bar: 200 nm.

SUPPLEMENTARY FIGURE S2 Different EpCAM and HER2 expression levels among human breast cancer cell lines (SK-BR-3, MDA-MB-453, MCF-7, MDA-MB-468) and normal basal mammary epithelial cell line (MCF-10A). **(A)** Percentage (%) of stained cells with phycoerythrin (PE)-labeled anti-EpCAM or HER2 antibodies (Red) compared with isotype control (black) from flow cytometry analysis. **(B)** Western blot analysis. β-actin was chosen as internal reference protein.

SUPPLEMENTARY FIGURE S3 HER2 immunostaining on the captured breast cancer cells by Pep@MNPs in a 106 U937 cells-contained system. **(A)** EpCAM and HER2 expression of human leukemia U937 cells from flow cytometry analysis. %, percentage of the cells positively stained with PE-labeled anti-EpCAM or HER2 (red) compared with isotype control (black). **(B)** Fluorescent graphs of the isolated breast cancer cells by Pep@MNPs in System 2 (1000 breast cancer cells + 106 U937 cells in 1 mL PBS) for each breast cancer cell line. The breast cancer cells were pre-stained with DAPI (blue, nuclei) and DiO (green, membrane), whereas U937 cells were only pre-stained with DAPI (blue, nuclei). The captured cells were then stained with Alexa Fluor®647-conjugated anti-HER2 (pink, HER2). Scale bar: 50 μm. **(C)** The mean fluorescent intensities (MFIs) from HER2 immunostaining on the captured breast cancer cells by Pep@MNPs in System 2. Fifty captured cells were analyzed for each breast cancer cell line. Data are presented as means ± SD. *****p* < 0.0001, Student’s t-test (unpaired) with Welch’s correction. **(D)** Correlation between the HER2 assessments of the captured cancer cells from System 1 (1000 breast cancer cells in 1 mL PBS) and System 2 (1000 breast cancer cells + 106 U937 cells in 1 mL PBS). Pearson’s correlation coefficient r = 0.9975 and ***p* = 0.0025 (two-tailed) indicated a positive, strong correlation at a significant level.

SUPPLEMENTARY FIGURE S4 Receiver operating characteristic (ROC) curve analysis of CTC enumeration by Pep@MNPs used to detect breast cancer. Fifty-two breast cancer patients and 10 healthy donors were totally included to achieve CTC enumeration by Pep@MNPs and immunocytochemistry. Healthy donors served as controls for assessing the specificity. The area under curve (AUC) (gray) was 0.875 (*p* = 0.0002). The black arrow indicates the cutoff with the maximized Youden index (i.e. 3 CTCs per 2.0 mL whole blood), which could be used to identify breast cancer patients (≥ 3 CTCs) sensitively and specifically. The detailed information from the ROC analysis is included in supplemental material (Supplementary Table S2).

SUPPLEMENTARY FIGURE S5 CTC number distribution in two subgroups of patients with non-visceral (n = 17) or visceral metastases (n = 35). Data are presented as scattered dots, together with means ± SD. *p* = 0.022, unpaired Student’s t-test with Welch’s correction.

SUPPLEMENTARY FIGURE S6 ROC analysis of the HER2(2+) CTC ratio used to differentiate the patients with various IHC-based HER2 status (IHC++ vs IHC+/0). Area under curve (AUC) was calculated as 0.755 (p = 0.021). Black arrow indicated the cutoff (12%) of the ratio of HER2(2+) CTCs with maximized Youden index, with moderate sensitivity (58.3%; 95% confident interval (CI), 32.0%–80.7%) and high specificity (100%; 95% CI, 81.6%–100%).

SUPPLEMENTARY FIGURE S7 Representative cases with a good concordance between the HER2 expression on CTCs and on tumor tissues at baseline, including patient #18 **(A)** and #51 **(B)**. Cell nuclei (blue) and HER2 proteins (brown) were visible due to immunochemical reactions on tumor tissues.

SUPPLEMENTARY FIGURE S8 Correlation analysis of pretherapeutic CTC enumeration with progression-free survival (PFS) **(A)** and overall survival (OS) **(B)** of non-HER2+ patients. Patients with various CTC enumeration (≥ 3 CTCs in pink; > 3 CTCs in gray) from 2.0 mL whole blood at baseline were compared. Log-rank test was used to assess the differences. *p* < 0.05, not significant (ns). N/A, median OS not yet reached.

SUPPLEMENTARY FIGURE S9 HER2 phenotyping of the enriched CTCs improves the prediction of survivals in HER2+ breast cancer. **(A,B)** Kaplan-Meier plots of progression-free survival (PFS) **(A)** and overall survival (OS) **(B)** in the patients with various CTC enumeration (≥ 3 CTCs in pink; > 3 CTCs in gray) from 2.0 mL whole blood at baseline. In PFS **(A)**, log-rank *p* = 0.168; in OS **(B)**, log-rank *p* = 0.219; both are > 0.05 (not significant, ns). **(C,D)** Kaplan-Meier plots

## Glossary

95% CI: 95% confidence interval
ATCC: American Type Culture Collection
AUC: area under curve
BSA: bovine serum albumin
CD45: leukocyte common antigen
CK: cytokeratin
CR: complete response
CTC: circulating tumor cell
*CTC*-HER2+: HER2 overexpression on CTCs
DAPI: 4’,6-diamidino-2-phenylindole dihydrochloride
DLS: dynamic light scattering
DMEM: Dulbecco’s Modified Eagle Medium
EpCAM: epithelial cell adhesion molecule
FBS: fetal bovine serum
FCM: flow cytometry
FDA: U. S. Food and Drug Administration
FISH: fluorescence *in situ* hybridization
HER2–: HER2-negative
HER2: human epidermal growth factor receptor 2
HER2+: HER2-positive
HR: hazard ratio
HRP: horseradish peroxidase
ICC: immunocytochemistry
IHC: immunohistochemistry
Ki67 LI: Ki67 labeling index
MEGM: mammary epithelial cell growth medium
MFI: mean fluorescent intensity
ORR: objective response rate.
OS: overall survival
PBS: phosphate buffer saline
PE: phycoerythrin
Pep@MNP: peptide-functionalized magnetic nanoparticles
PFA: paraformaldehyde
PFS: progression-free survival
PR: partial response
PVDF: polyvinylidene fluoride
RECIST: response evaluation criteria in solid tumors
ROC: receiver operating characteristic
TEM: transmission electron microscope
TKI: tyrosine kinase inhibitor
WBC: white blood cells
