# Supplementary figures and images for: HER2 status of CTCs by peptide-functionalized nanoparticles as the diagnostic biomarker of breast cancer and predicting the efficacy of anti-HER2 treatment
Source: Front Bioeng Biotechnol. 2022 Sep 28;10:1015295. doi: 10.3389/fbioe.2022.1015295 (PMC9554095; doi:10.3389/fbioe.2022.1015295)

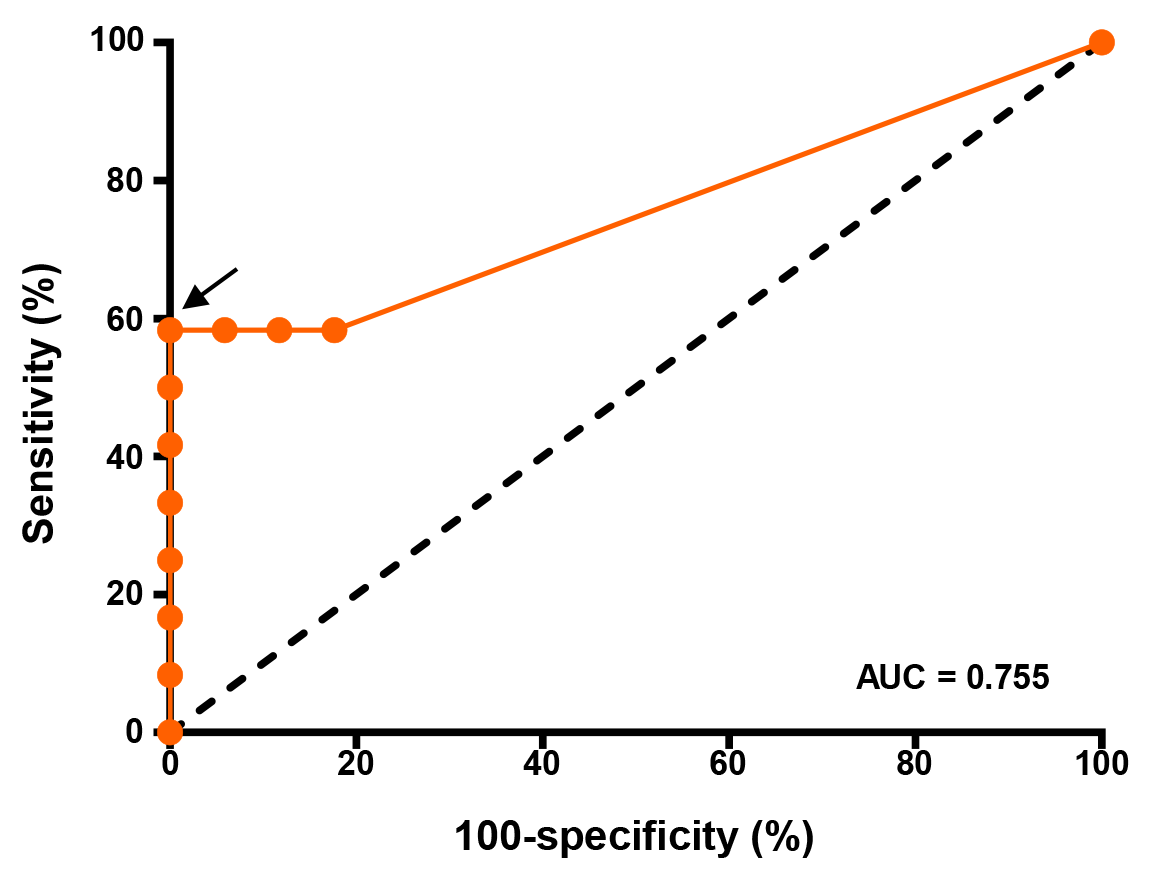

Supplement: Supplementary file 1 [file Image6.TIF]

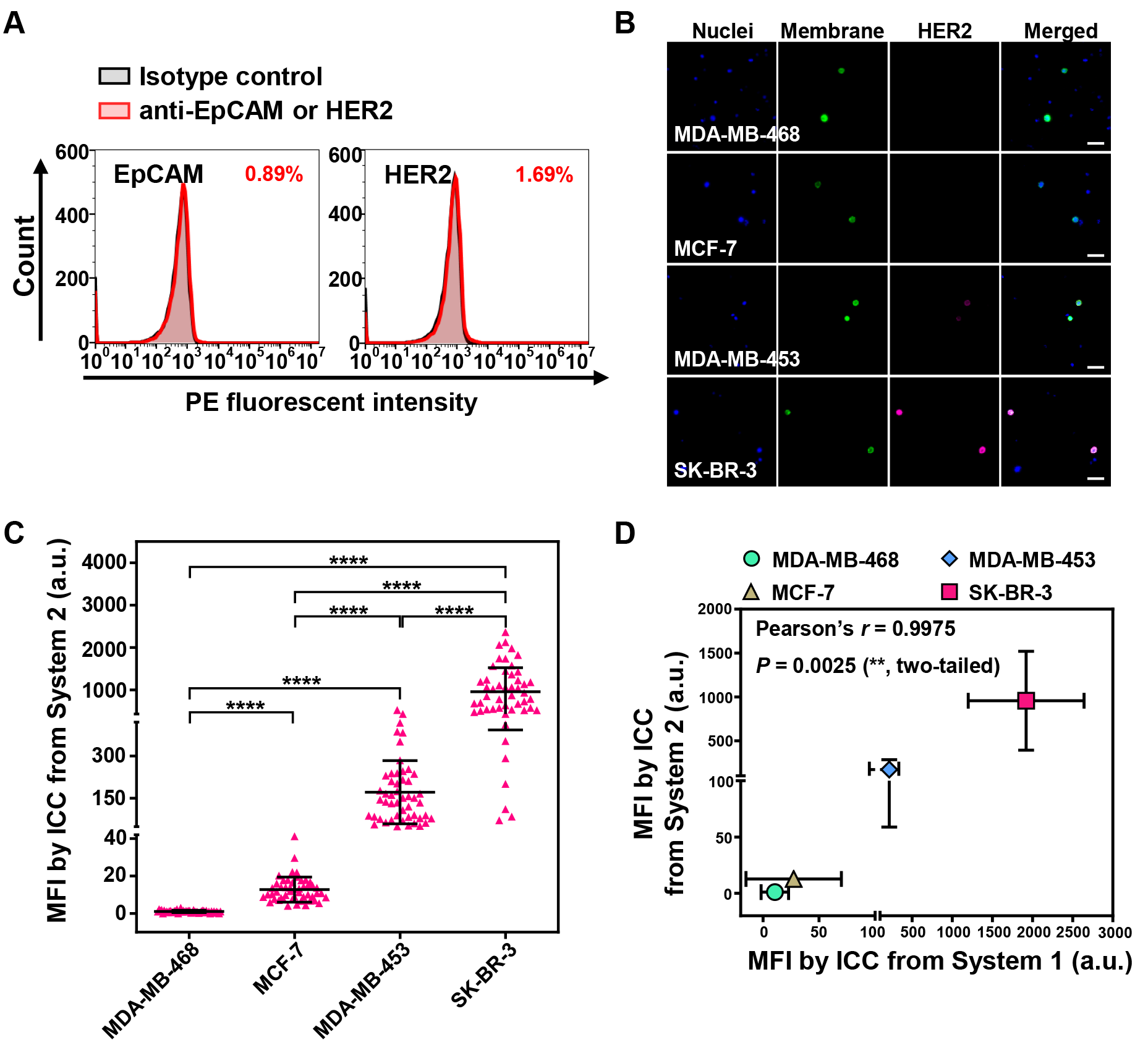

Supplement: Supplementary file 3 [file Image3.TIF]

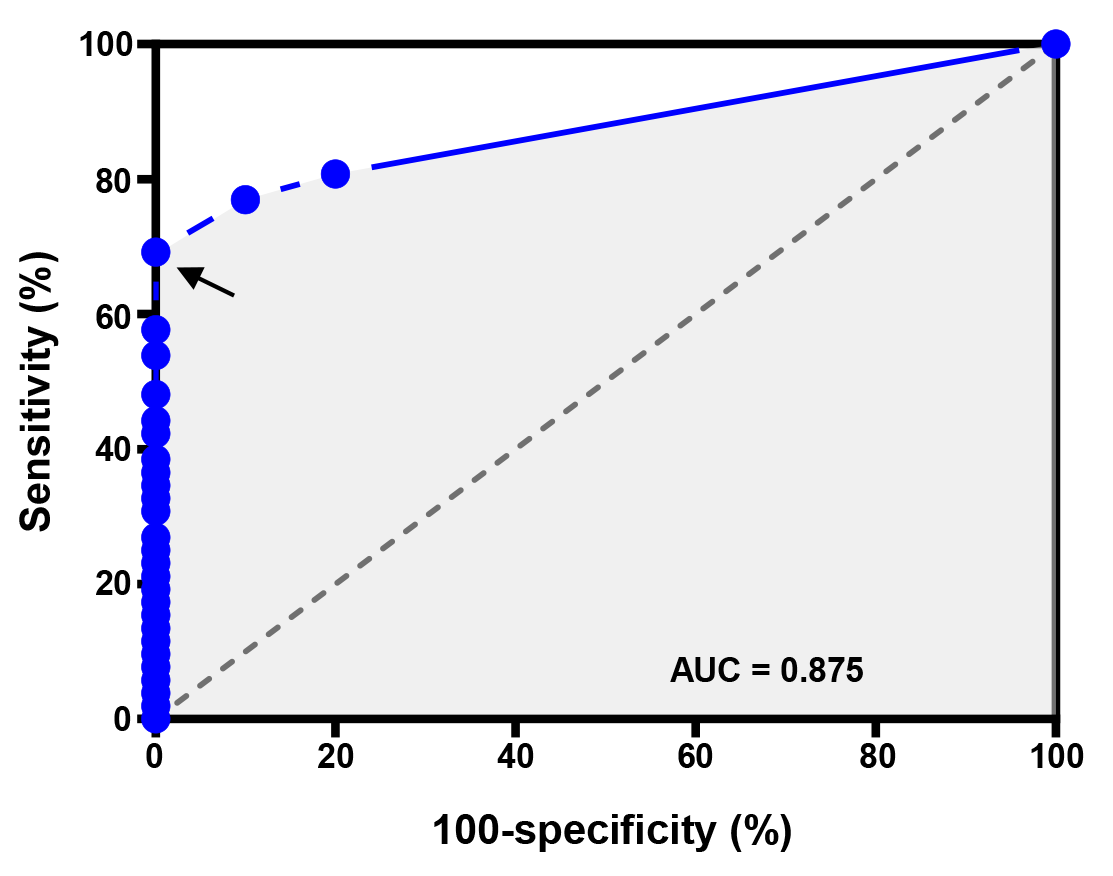

Supplement: Supplementary file 4 [file Image4.TIF]

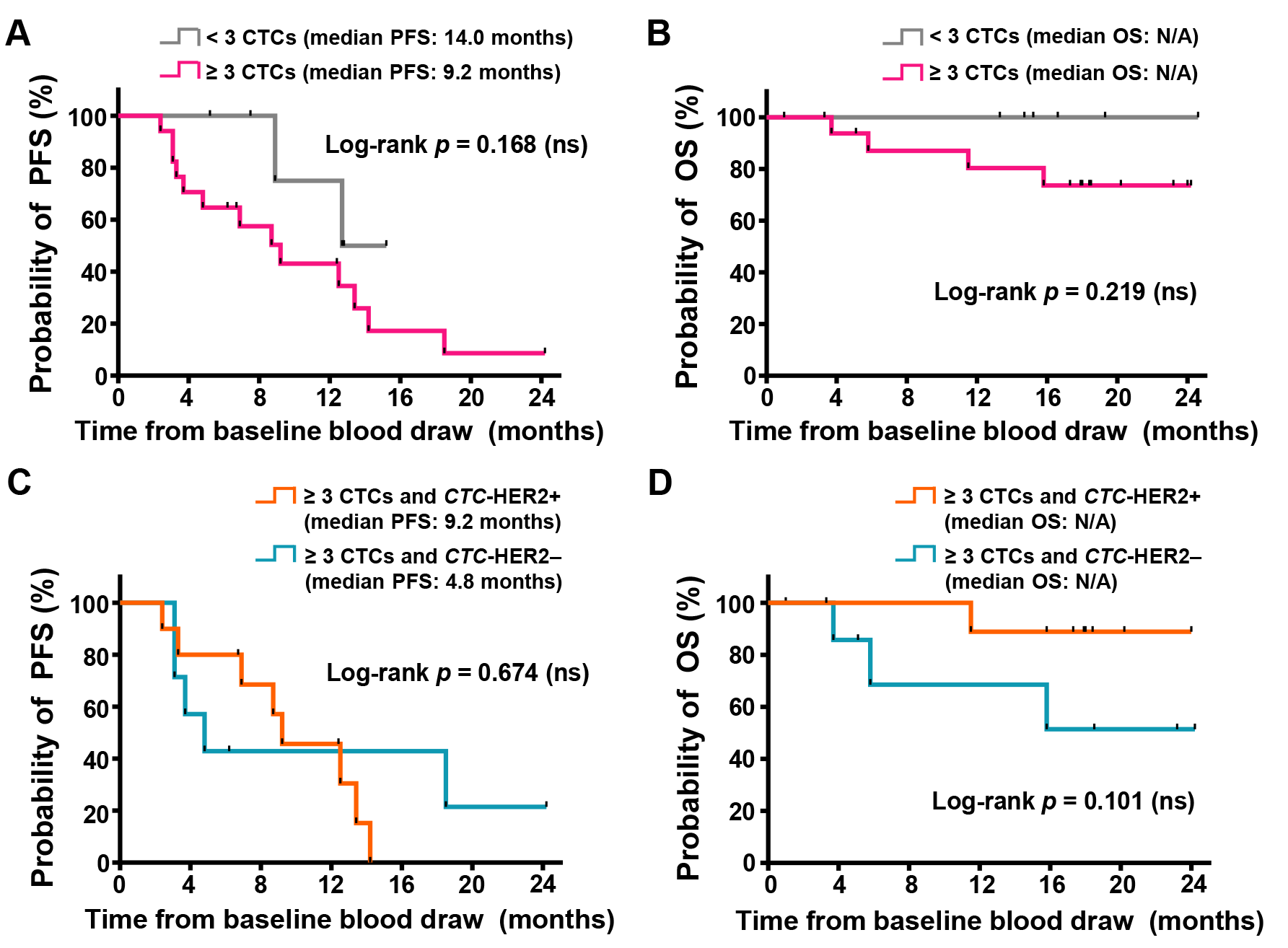

Supplement: Supplementary file 6 [file Image9.TIF]

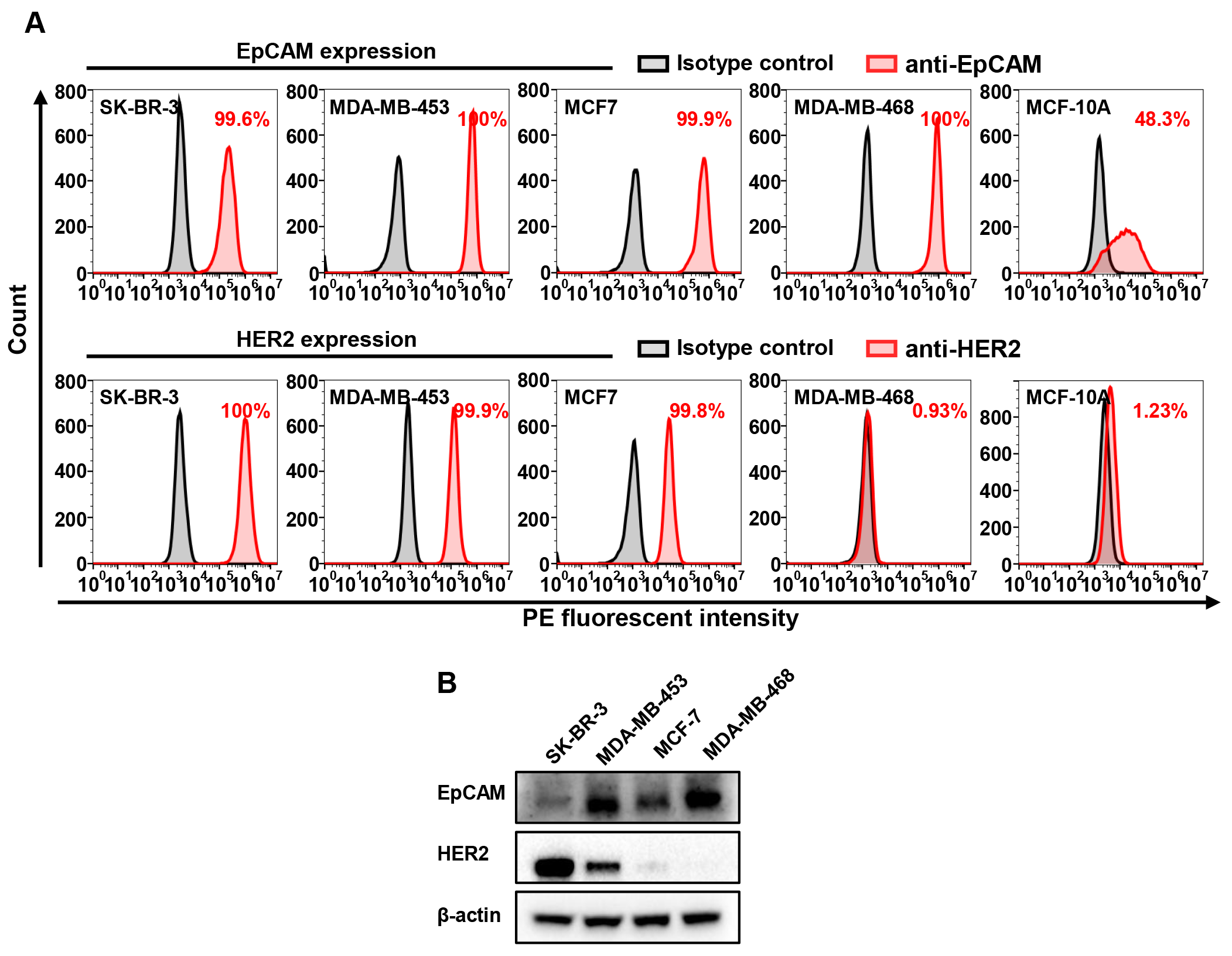

Supplement: Supplementary file 7 [file Image2.TIF]

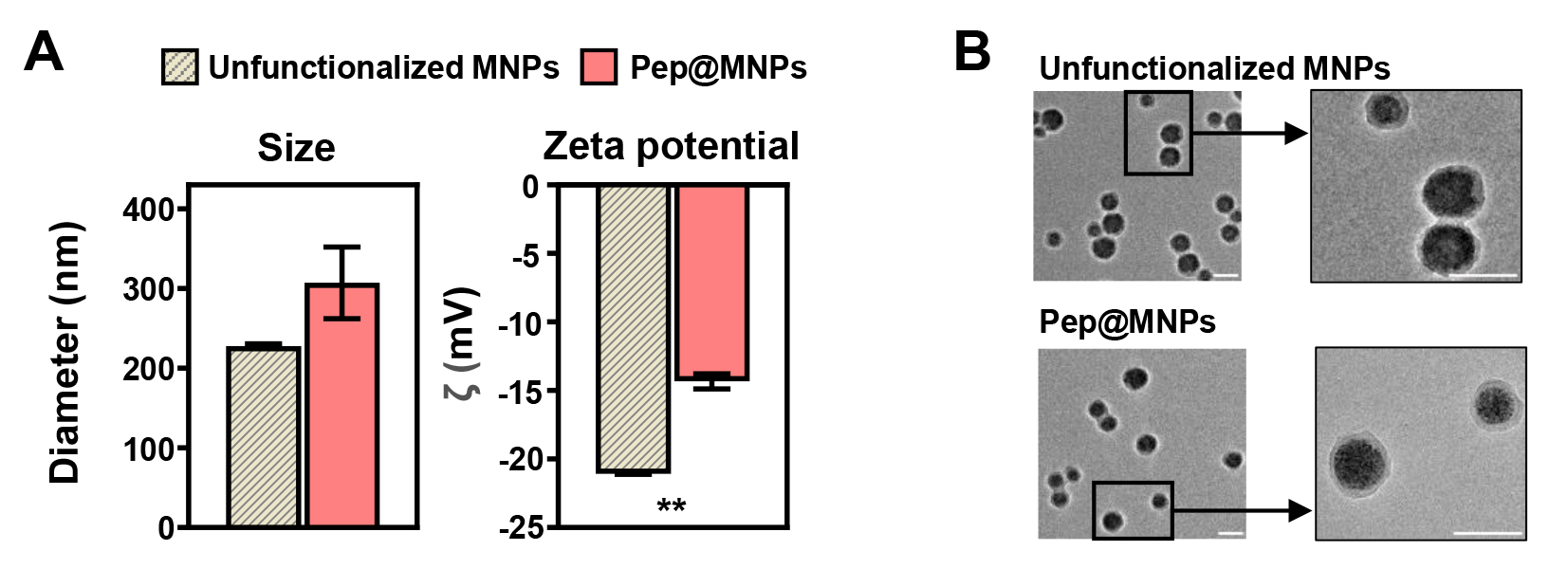

Supplement: Supplementary file 8 [file Image1.TIF]

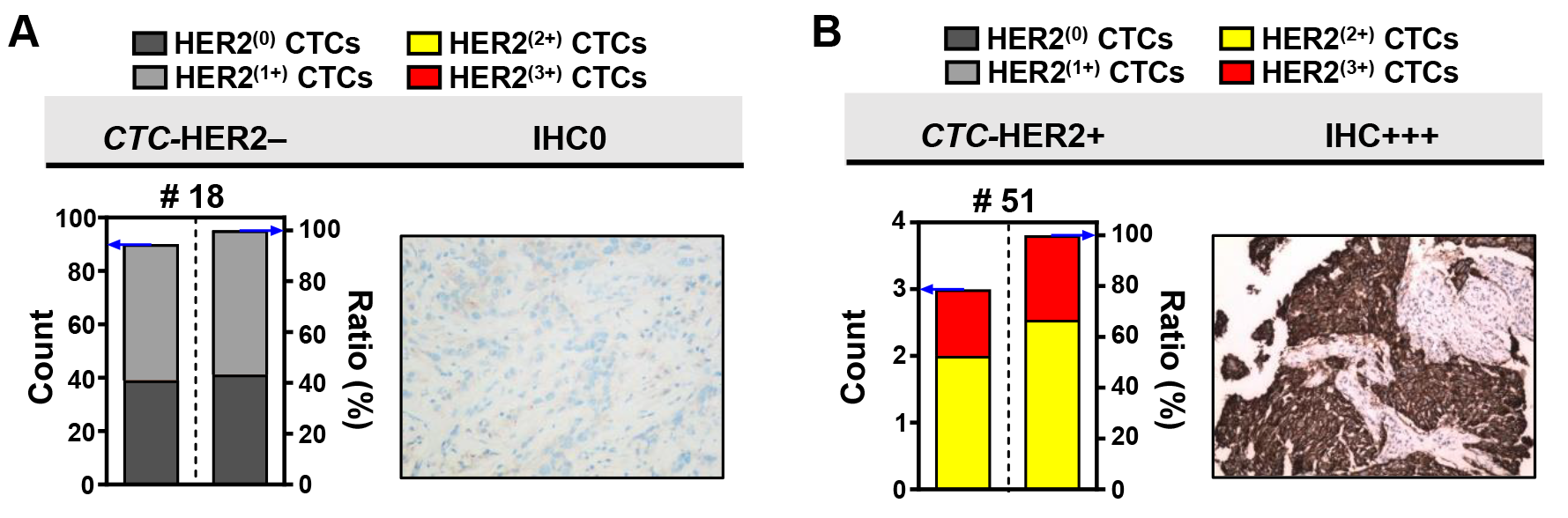

Supplement: Supplementary file 9 [file Image7.TIF]

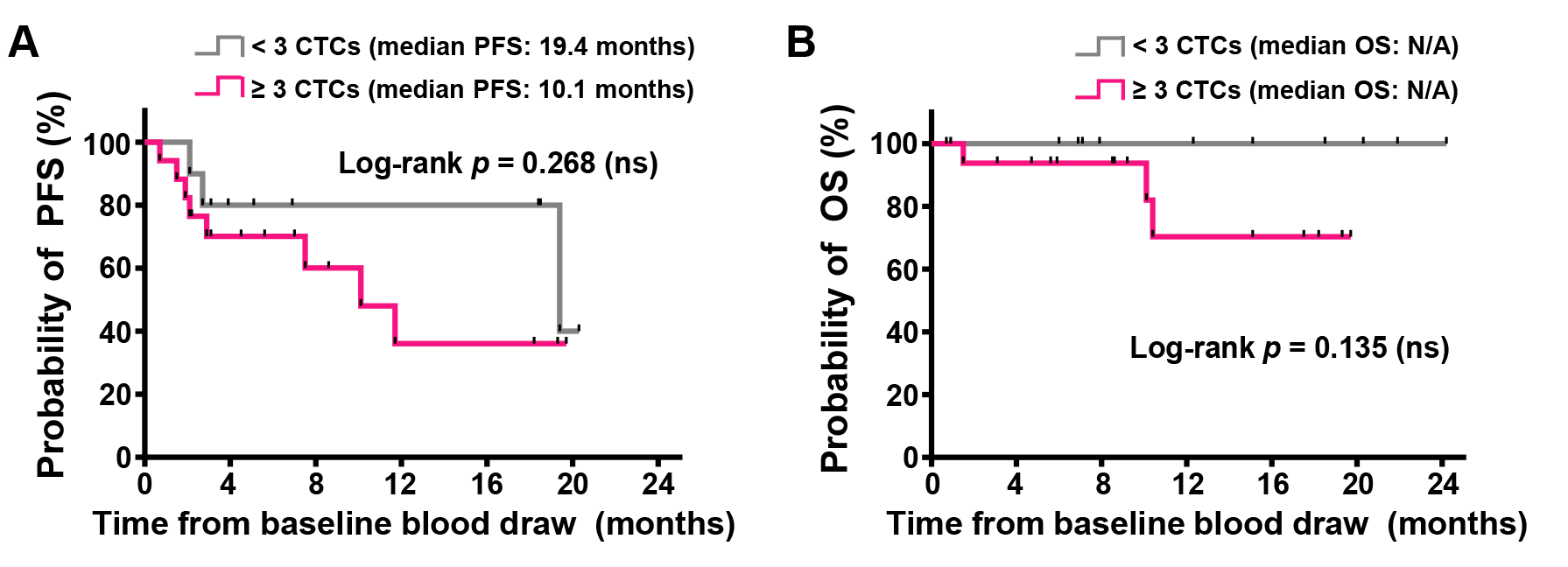

Supplement: Supplementary file 10 [file Image8.TIF]

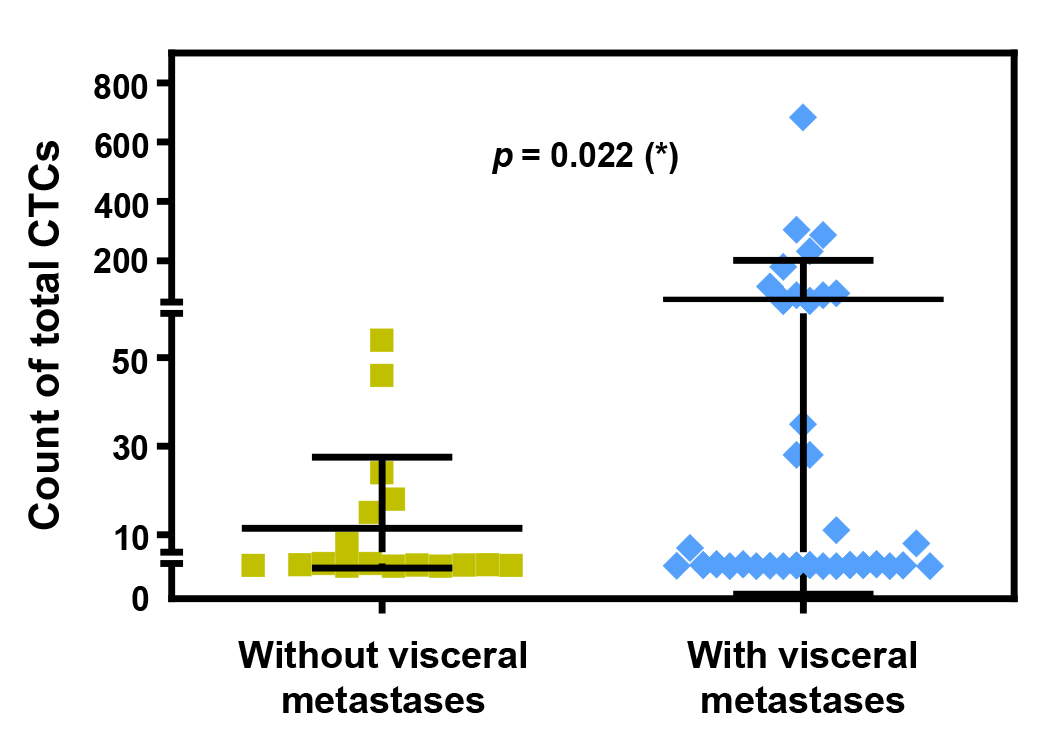

Supplement: Supplementary file 11 [file Image5.TIF]
