# Supplementary material for: HER2 status of CTCs by peptide-functionalized nanoparticles as the diagnostic biomarker of breast cancer and predicting the efficacy of anti-HER2 treatment
Source: Front Bioeng Biotechnol. 2022 Sep 28;10:1015295. doi: 10.3389/fbioe.2022.1015295 (PMC9554095; doi:10.3389/fbioe.2022.1015295)
Supplement: Supplementary file 2 [file Table1.PDF]

Supplementary TABLE S1. The clinical factors of 52 enrolled breast cancer patients and the testing results of 52 baseline blood samples and 13 second blood samples.

No.	Clinical No.	Age	Grading	Status	Visceral metastasis	HER2 status (IHC)	HER2 status (FISH)	CTC count at baseline (T0)	CTC-HER2 status (0 = negative; 1 = positive)	Time of first-line therapy	Best overall response	Time of progression or final follow-up visit	Progression (0 = lost or not progressed; 1 = progressed)	Duration from baseline blood draw to progression/final visit (progression-free survival PFS, months)	Time of death or final follow-up visit	Death (0 = lost or alive; 1 = death)	Duration from baseline blood draw to death/final visit (overall survival OS, months)	Time of second blood draw (T1)		Response at T1
#01	T001652258	42	G2	Recurrence	Yes	IHC+++	Unknown	0		2019-03-26	PR	2020-03-31	0	12.8	2021-03-22	0	24.6	2019-07-17	16.1	PR
#02	T001523676	56	G3	Recurrence	Yes	IHC+	Unknown	0		2019-03-26		2019-05-27	1	2.1	2020-03-30	0	12.3			
#03	0009710963	66	G1	Recurrence	Yes	IHC++	FISH–	0		2019-03-28		2020-10-31	1	19.4	2021-03-22	0	24.2			
#04	T001653800	69	G2	Recurrence	Yes	IHC+++	Unknown	0		2019-04-10	SD	2020-01-01	1	8.9	2020-11-09	0	19.3			
#05	T001679391	51	G2-3	Recurrence	No	IHC+	Unknown	0		2019-06-06		2019-12-30	0	6.9	2019-12-30	0	6.9			
#06	0009876966	36	G3	Recurrence	No	IHC+++	Unknown	0		2019-06-06	PR	2019-11-07	0	5.2	2020-10-14	0	16.6	2019-07-24	6.9	PR
#07	0009774322	60	G2	Recurrence	Yes	IHC0	Unknown	0		2019-06-05		2020-12-08	0	18.4	2021-03-22	0	21.9			
#08	T001694437	48	G3	Recurrence	No	IHC++	FISH–	0		2019-08-02		2021-03-22	0	20.3	2021-03-22	0	20.3			
#09	T001704690	54	G2	Recurrence	Yes	IHC+++	Unknown	0		2019-08-19	PR	2020-11-13	0	15.2	2020-11-13	0	15.2			
#10	T001707708	56	G1	Recurrence	Yes	IHC+++	Unknown	0		2019-09-03	PR	2020-09-07	1	12.7	2020-11-05	0	14.7			
#11	0009553569	56	G2	Recurrence	Yes	IHC0	Unknown	1	0	2019-03-28		2019-08-28	0	5.1	2019-11-19	0	7.9			
#12	0009874365	66	G3	Primary IV	No	IHC+	Unknown	2	0	2019-04-30		2020-10-16	0	18.5	2020-10-16	0	18.5			
#13	T001682991	69	Unknown	Recurrence	No	IHC0	Unknown	15	0	2019-06-25		2019-08-30	0	2.2	2020-09-21	0	15.1			
#14	T001658489	49	G2	Recurrence	No	IHC0	Unknown	46	0	2019-04-10		2019-06-10	1	2.1	2020-02-15	1	10.4			
#15	T001656192	44	G2-3	Recurrence	Yes	IHC0	Unknown	3	0	2019-03-27		2020-11-04	0	19.7	2020-11-04	0	19.7			
#16	0009858555	37	G3	Recurrence	No	IHC0	Unknown	6	0	2019-06-07		2019-07-31	1	1.9	2020-11-09	0	17.5			
#17	0009831596	38	G2	Recurrence	No	IHC0	Unknown	18	0	2019-03-20		2019-04-10	1	0.7	2019-04-10	0	0.7			
#18	0009872919	52	G3	Recurrence	Yes	IHC0	Unknown	90	0	2019-03-20		2019-10-30	1	7.5	2019-11-27	0	8.5			
#19	T001656927	55	G1	Recurrence	Yes	IHC0	Unknown	11	0	2019-04-09		2019-08-23	0	4.5	2019-08-27	0	4.7			
#20	0009703623	62	G2	Recurrence	Yes	IHC0	Unknown	3	0	2019-03-29		2020-01-27	1	10.1	2020-01-27	1	10.1			
#21	0009873470	61	G2	Recurrence	Yes	IHC++	FISH–	65	0	2019-04-03		2019-07-03	0	3.1	2019-07-03	0	3.1			
#22	T001647476	87	G3	Primary IV	No	IHC+	Unknown	24	0	2019-04-15		2019-07-10	1	2.9	2019-10-09	0	5.9			
#23	T001654638	57	G3	Recurrence	Yes	IHC+	Unknown	3	0	2019-03-28		2019-10-24	0	7.0	2019-12-27	0	9.2			
#24	T001660564	66	G3	Primary IV	No	IHC0	Unknown	4	0	2019-05-06		Lost			2019-05-08	0	0.9			
#25	0009770515	46	G2	Recurrence	Yes	IHC0	Unknown	5	0	2019-04-25		2019-05-28	1	1.5	2019-05-28	1	1.5			
#26	0009873629	62	G2	Primary IV	Yes	IHC++	FISH–	5	0	2019-03-20		2019-12-02	0	8.6	2019-12-02	0	8.6			
#27	T001654179	44	G3	Recurrence	No	IHC0	Unknown	54	0	2019-03-22		2019-09-05	0	5.6	2019-09-05	0	5.6			
#28	T001654655	64	G2	Recurrence	Yes	IHC0	Unknown	304	0	2019-03-26		2020-10-20	0	19.3	2020-10-20	0	19.3			
#29	0009798832	53	G3	Recurrence	Yes	IHC0	Unknown	113	0	2019-03-21		2020-09-17	0	18.2	2020-09-17	0	18.2			
#30	T001704191	56	G3	Recurrence	No	IHC++	FISH–	3	1	2019-08-26		2020-07-24	1	11.7	2020-11-05	0	15.1			
#31	T001695722	56	G2	Recurrence	No	IHC++	FISH–	2	1	2019-08-02		2019-10-25	0	3.1	2020-10-20	0	15.1			
#32	T001693130	56	G3	Recurrence	Yes	IHC++	FISH–	2	1	2019-07-08		2019-09-27	1	2.7	2020-02-05	0	7.1			
#33	T001451043	54	G2	Recurrence	Yes	IHC++	FISH–	1	1	2019-07-11		2019-10-30	0	3.9	2019-12-31	0	6.0			
#34	0009824914	52	G3	Recurrence	No	IHC+++	Unknown	4	0	2019-04-15	SD	2021-03-22	0	24.2	2021-03-22	0	24.2	2019-07-09	12.1	SD
#35	0009800076	55	G2	Recurrence	Yes	IHC+++	Unknown	84	0	2019-05-14	Lost	Lost			2019-06-10	0	1.0			
#36	0009874871	66	G3	Recurrence	Yes	IHC+++	Unknown	232	0	2019-06-03	SD	2019-10-08	1	4.8	2020-08-31	1	15.8	2019-07-17	6.3	SD
#37	0009861810	38	G1	Recurrence	Yes	IHC++	FISH+	64	0	2019-05-17	Lost	2019-09-05	1	3.7	2019-09-05	1	3.7			
#38	0009874941	48	G2	Primary IV	No	IHC+++	Unknown	8	0	2019-04-19	SD	2019-10-23	0	6.2	2020-10-26	0	18.5	2019-07-15	12.4	SD
#39	T001667018	59	G3	Recurrence	Yes	IHC+++	Unknown	28	0	2019-04-25	SD	2020-10-30	1	18.5	2021-03-22	0	23.2	2019-07-26	13.1	SD
#40	0009872756	60	G2	Primary IV	Yes	IHC+++	Unknown	83	0	2019-04-01	PD	2019-06-22	1	3.1	2019-08-19	0	5.1			
#41	T001714019	45	G2	Recurrence	Yes	IHC+++	Unknown	2	0	2019-09-29	PR	2020-04-29	0	7.5	2020-10-21	0	13.3			
#42	T001456810	42	G2	Recurrence	Yes	IHC+++	Unknown	287	0	2019-05-16	SD	2019-08-09	1	3.1	2019-10-31	1	5.8	2019-08-09	12.1	PD
#43	T001664903	48	G2	Recurrence	Yes	IHC++	FISH+	8	1	2019-05-21	PD	2019-07-31	1	2.4	2020-11-09	0	17.9			
#44	0009859373	47	G2	Recurrence	No	IHC++	FISH+	6	1	2019-07-29	SD	2020-08-30	1	13.4	2021-03-22	0	20.2			
#45	T001716887	60	G3	Primary IV	Yes	IHC++	FISH+	5	1	2019-09-24	PR	2020-06-15	1	9.2	2021-03-22	0	18.4			
#46	T001594057	69	G3	Recurrence	No	IHC+++	Unknown	3	1	2019-05-05	PR	2019-11-06	0	6.7	2020-08-05	0	15.8	2019-07-09	9.3	PR
#47	0009857570	63	G2-3	Recurrence	Yes	IHC+++	Unknown	683	1	2019-05-14	PR	2020-07-09	1	14.2	2020-10-30	0	18.0	2019-07-06	7.6	PR
#48	0009769973	33	G3	Recurrence	Yes	IHC++	FISH+	179	1	2019-03-22	PR	2019-12-06	1	8.7	2020-08-21	0	17.3	2019-07-11	15.9	PR
#49	0009817831	32	G3	Recurrence	Yes	IHC+++	Unknown	35	1	2019-04-02	PR	2020-04-09	0	12.4	2021-03-22	0	24.0	2019-07-26	16.4	PR
#50	0009894260	39	G3	Recurrence	Yes	IHC++	FISH+	28	1	2019-09-06	PR	2020-03-24	1	6.9	2020-08-10	1	11.5	2019-10-22	6.6	PR
#51	0009895857	42	G3	Primary IV	Yes	IHC+++	Unknown	3	1	2019-11-28	SD	2020-02-18	1	3.3	2020-02-18	0	3.3			
#52	T001676330	68	G3	Recurrence	Yes	IHC+++	Unknown	7	1	2019-05-29	SD	2020-05-28	1	12.5	2020-11-09	0	18.0	2019-07-15	6.7	SD
