# Supplementary material for: HER2 status of CTCs by peptide-functionalized nanoparticles as the diagnostic biomarker of breast cancer and predicting the efficacy of anti-HER2 treatment
Source: Front Bioeng Biotechnol. 2022 Sep 28;10:1015295. doi: 10.3389/fbioe.2022.1015295 (PMC9554095; doi:10.3389/fbioe.2022.1015295)
Supplement: Supplementary file 5 [file Table2.pdf]

Supplementary TABLE S2 Clinical effect size of CTC enumeration used to differentiate between breast cancer patients and healthy donors from receiver operating characteristic (ROC) curve analysis.

	**CTC enumeration**
AUC, *95% CI*	0.875, *0.789 to 0.961*
Std. Error	0.044
p value	0.0002
Sensitivity, *95% CI*	69.2%, *55.7% to 80.1%*
Specificity, *95% CI*	100%, 72.3% *to 100%*

CTC, circulating tumor cells; AUC, area under the curve; Std. Error, standard error; 95% CI, 95% confidence interval; Sensitivity, the percentage of patients detected with ≥ 3 CTCs in 2.0 mL whole blood in 52 breast cancer patients; Specificity, the percentage of healthy donors detected with < 3 CTCs/2.0 mL whole blood in 10 healthy donors.
